# Individual threat-relevance accelerates belief-updating in conditioned hallucinations

**DOI:** 10.1038/s41598-026-52299-9

**Published:** 2026-06-13

**Authors:** Joshua M. Martin, Victoria L. Fisher, Anna-Lena Eckert, Albert Powers, Philipp Sterzer

**Affiliations:** 1 Berlin School of Mind and Brain, Faculty of Philosophy, Humboldt-Universität zu Berlin, Berlin, Germany; 2 Department of Psychiatry and Psychotherapy, Charité–Universitätsmedizin Berlin, Campus Charité Mitte, Berlin, Germany; 3 Yale University School of Medicine and the Connecticut Mental Health Center, New Haven, CT USA; 4 Department of Neuroscience, Columbia University, New York, NY USA; 5 Department of Psychology, Theoretical Cognitive Science Group, Philipps-Universität Marburg, Marburg, Germany; 6 Department of Psychiatry (UPK), University of Basel, Basel, Switzerland

**Keywords:** Neuroscience, Psychology, Psychology

## Abstract

Threat-related learning is implicated in psychopathological hallucinations; however, its role in experimentally-induced hallucinations remains unclear. Here, we investigated how threat-relevant stimuli (spiders vs. flowers) influence conditioned hallucinations (i.e., illusory auditory tones triggered by learned associations with images). We found that, for conditioning with spiders (but not flowers), higher spider-phobia was related to time-dependent variations in conditioned hallucinations: increasing in early phases (where spiders reliably predicted tones) and decreasing in later phases (as this predictive relationship weakened). These tendencies were related to accelerated belief-updating in a hierarchical Gaussian filter model and shifts in participants’ internal criterion within a signal detection framework. Additionally, trait anxiety and hallucination-proneness were inversely related to conditioned hallucinations and reliance on prior beliefs, but did not significantly vary according to image condition. Unlike the theorized role of threat in developing rigid priors and clinical hallucinations, our findings indicate that individual threat-relevance sensitises hallucination-related beliefs to shifting stimulus contingencies.

**Supplementary Information:**

The online version contains supplementary material available at 10.1038/s41598-026-52299-9.

## Introduction

Hallucinations can be defined as percepts that occur in the absence of corresponding external sensory stimuli. Despite extensive research, they are challenging to treat in psychiatric illness, where they can be a source of significant distress. In the last two decades, theories grounded in Bayesian inference, such as predictive processing^1–4^, have gained traction as models of the computational principles underlying hallucinations and related psychopathologies. These accounts build on Helmholtz’s idea^5^ that perception arises from inferential processes, where the brain weighs sensory evidence and prior beliefs to infer hidden causes of sensory input. Imbalances in this weighting process are thought to lead to dysfunctional inference and psychopathology. For example, over-weighted priors are theorized to underlie the generation of hallucinations across medical disorders and in the general population^2,3,4,6–8^.

One main line of evidence for this ‘over-weighted prior’ view comes from findings based on the conditioned hallucinations paradigm. This method was pioneered by Ellson during the 1940’s^9^, and, in recent years, revisited with neuroimaging^10–12^ and computational modelling^10,11,13–15^. The method involves inducing hallucinations in one modality (e.g., hearing a tone) by repeatedly pairing it with a sensory cue in another modality (e.g., seeing an image). Over time, this associative learning leads participants to report hearing tones in response to seeing the visual cue, even when no such auditory stimulus was presented^9^. Importantly, these hallucinated tones engage tone-responsive brain regions^10^, suggesting they reflect genuine percepts rather than purely cognitive or decisional processes. Several studies have found that individuals who report hallucinatory experiences in everyday life are more susceptible to this effect^10–15^ and, in line with the role of over-weighted priors, tend to rely more heavily on their prior beliefs in formulating perceptual decisions during the task^10,11,13,14,16^. It remains unclear the extent to which this effect is modality-specific; however, a visual variant of the task found increased susceptibility among individuals reporting either visual or auditory hallucinations, suggesting it may generalize across modalities^15^.

While various factors influence conditioned hallucinations^17^, one overlooked aspect is the role of threat-relevance. Threat-relevance refers to stimuli that are ontogenetically or phylogenetically linked to fear or danger. For example, in humans, spiders can be considered an evolutionary threat due to their venomous bites^18^. Such stimuli are perceptually prioritised by human subjects who more rapidly detect them in visual searches^19^, more rapidly and accurately detect them in two-alternative forced-choice tasks^20^, and gain preferential access to visual awareness^21,22^. When they are task irrelevant, spiders are still captured by attention^23^ and are more resistant to attentional disengagement^24,25^. This prioritisation appears to result from a combination of top-down expectation and bottom-up stimulus-driven features^20,26^. Spider phobia, affecting 2.7–6.1% of the population^27^, intensifies these effects by further impairing attentional disengagement from spider stimuli^24,25,28^ and increasing their likelihood of reaching perceptual awareness^21,22,29^.

Threat-related learning is theoretically implicated in the development of hallucinations in certain psychopathologies. Traumatic experiences, often involving life-threatening events, are a well-established contributor to hallucinations in PTSD^30^ and are increasingly recognized as a risk factor for hallucinations in schizophrenia^31^ and in the general population^32^. Dose-dependent relationships between the amount of trauma and hallucinations suggest a causal relationship^32,38^. Hallucinatory content in conditions like schizophrenia and PTSD is also often negative and threatening^33,34^, frequently reflecting direct or indirect links to earlier traumatic events^35–38^. Other conditions, such as Parkinson’s disease and Ekbom syndrome, can produce hallucinations with thematic links to evolutionary concerns, such as feeling spiders crawling over one’s hair and body^39,40^ or seeing and feeling bugs biting and crawling on one’s skin^41^. Additionally, individuals prone to hallucinations exhibit heightened interpretative biases toward socially meaningful^42^ and threatening^43^ stimuli, implicating biased expectations for perceiving biologically relevant events^44^. Finally, some theories of hallucinations based on predictive processing directly implicate overly precise threat-related priors in hallucinations^6,7,44,45^. Within this framework, hallucinations can be conceptualised as by-products (‘false alarms’) of a perceptual system optimized for detecting and responding to potential threats^44,46^.

In this preregistered study (see 10.17605/OSF.IO/SRHQD for protocol), we investigated how threat-relevance influences conditioned hallucinations by engendering hallucinations of auditory tones based upon the presence of images of either spiders or flowers. We chose spiders as our stimuli of interest, since they reliably evoke affective responses in human observers^21^ and spider-phobia is known to vary widely in the general population^27^. Based on the existing literature, we hypothesized that threat-relevance could plausibly impact conditioned hallucinations in one of two directions: first, due to the theorized role of threat-related learning in overly-weighted priors and hallucinations, spider images may enhance associative learning in our task, intensifying conditioned hallucinations. Second, given that images of spiders are harder to attentionally disengage from, threat-relevant stimuli may instead capture attention away from the target stimulus during conditioning (akin to a distraction effect), which may impair the formation of associations and weaken conditioned hallucinations. We additionally hypothesized that these effects would be modulated by individual differences in spider phobia, trait anxiety and hallucination-proneness. Finally, we used a hierarchical Gaussian filter (HGF) model^4,11,13–15,47^ to test our final hypothesis that observed changes in conditioned hallucinations would be related to the weighting of prior beliefs during the task.

## Materials and methods

### Participants

Participants were recruited via the online platform Prolific, with the study coordinated through Yale’s REDCap system^48^. Eligibility criteria included ages 18–65, normal or corrected vision, a 100% Prolific approval rate, and at least 10 prior submissions on the platform. The age range was chosen to match previous online studies using this paradigm^14^; to our knowledge, age-related effects on conditioned hallucinations have not yet been evaluated. From an initial sample of 549 participants, 102 were excluded, leaving a final sample of 447 (see Supplementary Material Sect. 1 for details). The target sample size was chosen to closely match that of a previous large online study using a similar conditioned hallucinations paradigm and between-subjects design^14^. Exclusions were based on preregistered metrics of poor task adherence (*n* = 65), clinical diagnoses or substance use during testing (*n* = 23), technical issues (*n* = 5), missing questionnaire data (*n* = 4), reported age over 65 (*n* = 2), and suspicious automated-like responses (*n* = 3). Subjects gave informed consent and were financially compensated for their participation. The study was approved by the Charité ethics committee and conformed to the principles laid out in the Declaration of Helsinki.

### Procedure

**Fig. 1:**
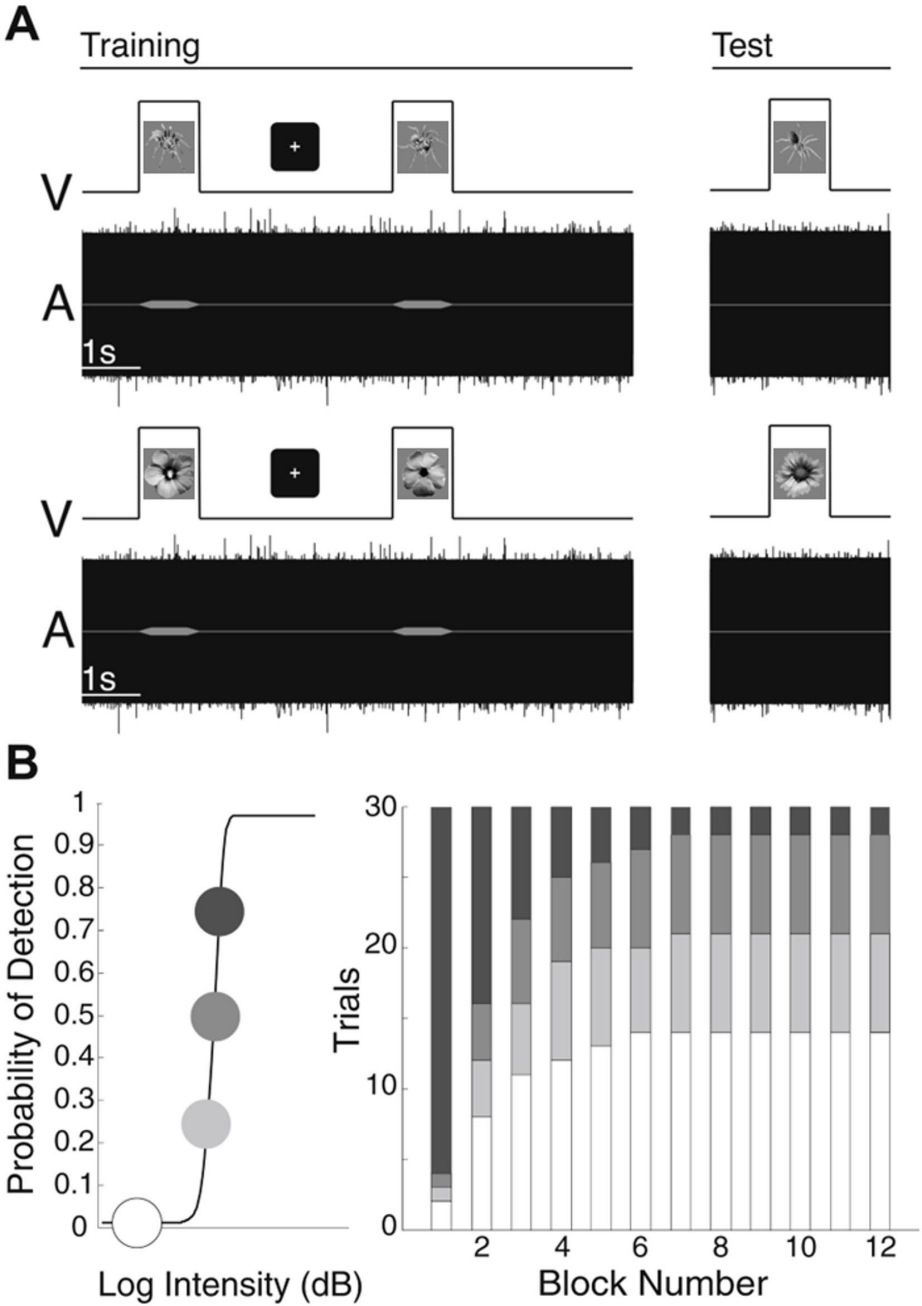
Illustration of the conditioned hallucinations task stimuli and design. (**a**) Participants learn to associate the presence of images with tones (left) and then are subsequently tested on this relationship (right). (**b**) Participants undergo stimulus thresholding to determine tone intensity levels for different trial types (left), which vary non-linearly in their distribution over time/experimental blocks (right).

Participants performed an initial QUEST thresholding procedure, consisting of two interleaved staircases of 40 trials each to determine subjective thresholds at which they were 75% likely to report detecting an auditory tone embedded in white noise. A Weibull psychometric function was then fit to determine the intensity levels at which they are estimated to be 25% and 50% likely to report hearing a tone. Thresholding was conducted in the absence of concurrent presentation of experimental imagery (i.e., spiders or flowers) to avoid any conditioning or habituation effects that may impact behavior in the main experiment. Instead, responses were cued by a color change in the fixation point occurring after the presentation of the tone on each trial. During the experimental phase, participants implicitly learnt to associate images and tones, and their learning of this relationship was evaluated over 360 trials across 12 experimental blocks (see Fig. 1a). Participants were randomly assigned to be conditioned with either spider images or flower images. A between-subjects design was used to align with previous online implementations of the paradigm^14^ and to avoid the possible difficulties in recruiting the same online participants across multiple sessions. Each trial involved presenting an image (spider or flower) concurrently with a tone embedded in white noise at one of three intensity levels (75%, 50%, 25% thresholds), or with only white noise (‘no-tone’ trials). Trial proportions varied non-linearly, with more high-intensity trials in early phases and more low-intensity and no-tone trials in later phases (see Fig. 1b). Participants reported whether they heard a tone (yes/no) and held their keypress to indicate their confidence on a 1–5 visual-analog scale. Conditioned hallucinations were defined as reporting a ‘yes’ response on a no-tone trial.

Following the main task, participants completed some basic demographic questions, answered three psychometric scales measuring variables of interest (spider-phobia^49^, trait anxiety^50^, and hallucination-proneness^51^), and provided subjective ratings of arousal, valence, and prototypicality in relation to the 14 experimental images employed in the study. Participants were then thanked and compensated for their participation. The median completion time of the entire study was 59 min.

### Materials

#### Experimental stimuli

The experimental images used were obtained from a previous study by Schmack et al.^21^ who investigated the neural basis of affective salience. In total, 14 images (7 spiders and 7 flowers) were selected for visual stimuli from an initial sample of 29 (15 flowers, 14 spiders). The selection was based on an optimization algorithm to minimize the difference in variance of the mean and standard deviation of spatial frequency in the two image sets (see Supplementary Material Sect. 2). This was done to minimize variability in low-level features that may contribute to differences in stimulus conditioning between the two groups. The final images were converted to grayscale, equalized in terms of root mean square contrast (57.26), and histogram matched using the SHINE toolbox^52^(see Supplementary Material Sect. 3.1 for examples). Matching the images in terms of chromatic information and luminance statistics is common in studies using threat-related stimuli^22–24^ and helps minimize differences in low-level visual features that could otherwise confound learning-related effects. For the auditory stimuli, a 1250 Hz sine-wave pure tone of 300ms duration was presented at a 70-dB sound pressure level.

#### Self-report measures and rating scales

We used three self-report measures as experimental predictors: (1) hallucination proneness as measured by the Launay-Slade Hallucination Scale Extended (LSHS^51^, a 16-item widely used measure of hallucinations across both clinical and non-clinical populations); (2) spider phobia as measured by the Spider Distress Scale (SDS^49^, a 17-item validated measure of spider fear and disgust suitable for the general population); and (3) trait anxiety as measured by the trait version of the Spielberger state-trait anxiety inventory (STAI^50^, a 20-item widely used measure of general dispositional anxiety). For image ratings, participants were presented with all 14 images (7 spiders, 7 flowers) and indicated their arousal (0, + 10), valence (-5, + 5) and prototypicality (0, + 10) ratings for each image on a visual-analog scale (see Supplementary Material Sect. 3.2 for details). Valence was measured on a bipolar scale (− 5 to + 5) to capture the opposing dimensions of pleasantness and unpleasantness, whereas arousal was measured on a unipolar scale (0–10) to capture the intensity of arousal. For the demographic questionnaire, participants reported their age, gender, and completed level of education.

### Statistical analyses

Statistical analyses were conducted in R (version 4.4.1^53^), while the HGF was carried out in MATLAB using the TAPAS toolbox (github.com/translationalneuromodeling/tapas). A comprehensive overview of model diagnostics can be found in Supplementary Material Sect. 5-8. Model diagnostics were performed using the DHARMa^54^ and performance^55^ packages. For all models, time was operationalised using block number (1–12) rather than trial number (1-360), as trial-level modeling was not computationally feasible. Effect sizes are reported as standardized beta coefficients (β) along with their 95% Wald confidence intervals.

The inferential analyses are divided into four main sections, corresponding to the structure of the Results section. This plan includes both preregistered and exploratory analyses; exploratory analyses are explicitly noted in their subheading. We describe each of these sections in turn.

### Behavioral outcomes: predicting conditioned hallucination frequency and confidence

We used generalized mixed effects models (GLMMs) to model how our experimental variables relate to the frequency and confidence of conditioned hallucinations (i.e. reports of hearing tones on no-tone trials). To model the frequency of reported conditioned hallucinations (‘response model’), we employed a GLMM with a binomial outcome to predict the probability of responding ‘yes’ on no-tone trials. To model the confidence in conditioned hallucinations (‘confidence model’), we employed a GLMM with a Gaussian outcome (or simply a linear mixed effects model) to predict reported confidence for ‘yes’ reports on no-tone trials.

Fixed and random effects were selected by a top-down approach using hierarchical model comparison by iteratively removing effects according to their log-likelihood ratios^56^(see Supplementary Material Sect. 5.1 for details). For random effects, we considered random intercepts and random slopes (over time) for each participant. Random intercepts account for interindividual differences in baseline response and confidence levels, whereas random slopes account for interindividual differences in the change in these outcomes over time. For fixed effects, we included group (spider images vs. flower images), time (experimental blocks 1–12), spider phobia (SDS score), hallucination proneness (LSHS score) and trait anxiety (STAI score), as well as their interactions. In line with our preregistration, we did not consider interaction effects between trait variables to avoid estimating four-way interactions. We deviated from our preregistered analysis plan by modelling the three trait-based fixed effects and their interactions within the same model, rather than testing them in separate models. This approach was computationally feasible and enabled estimation of each trait’s unique contribution to the outcome by accounting for shared variance among other trait predictors.

### Computational outcomes: relating predictors to the weighting and use of prior beliefs

**Fig. 2:**
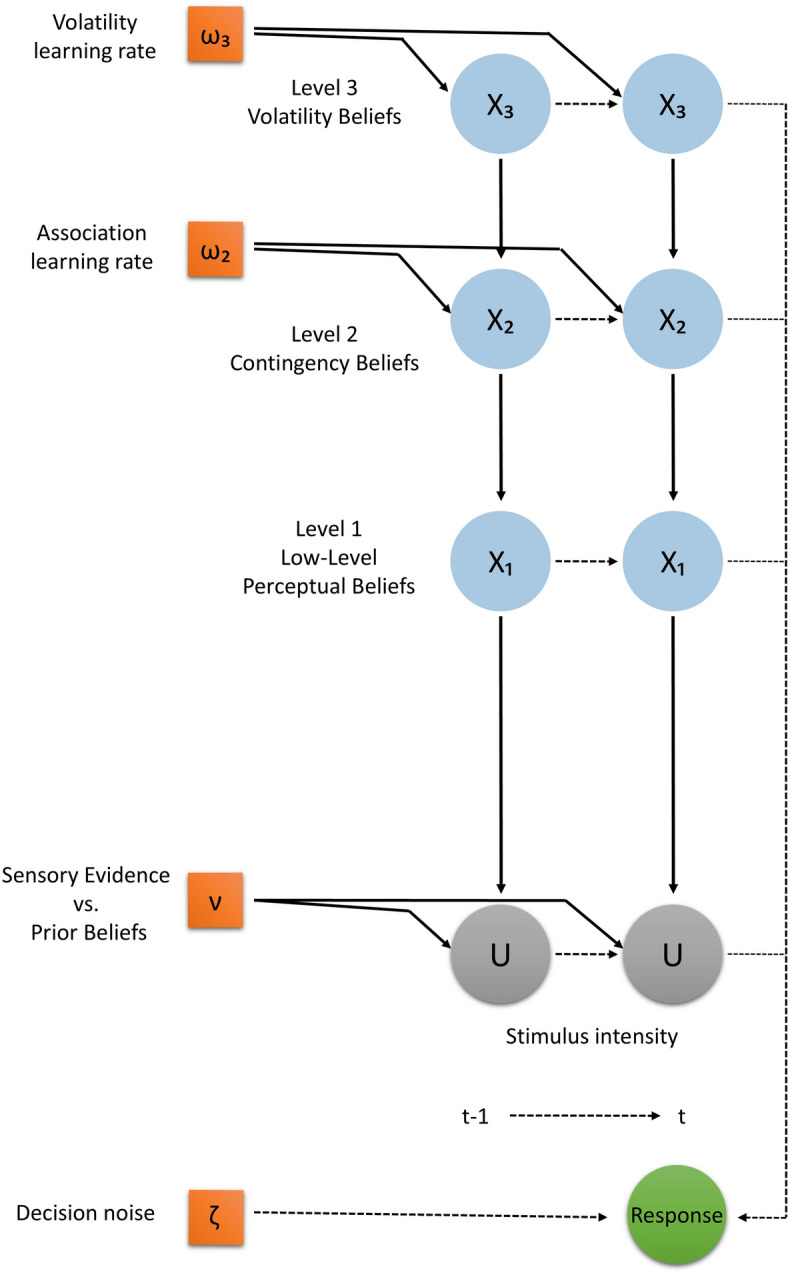
Hierarchical Gaussian Filter (HGF) model. We fitted a three-level HGF to trial-wise responses to model hierarchical belief updating about tone presence (X₁), tone–image associations (X₂), and the volatility of these associations (X₃). The model additionally estimates learning rates (ω₂, ω₃), the relative weighting of prior beliefs and sensory input (*v*), and decision noise (ζ).

To evaluate how our observed effects relate to the role of prior beliefs and belief-updating on conditioned hallucinations, a three-level HGF model^47^ was fitted to decompose perceptual decision-making behaviors into different underlying computational parameters (see Fig. 2). Unlike the behavioral analyses, which focused on no-tone trials, the HGF was fitted to the entire sample. The model consists of three levels representing (1) belief in tone presence (X1), (2) belief in tone-image associations (X2), and (3) belief in the volatility of these associations (X3). In addition to these belief weights, the model estimates, for every participant, parameters relating to the overall reliance on prior beliefs relative to sensory evidence (*v*), evolution rates encoding the transition probabilities between levels one and two (ω₂) and two and three (ω₃), and decision noise derived from a separate response model (ζ). This model has been specifically tailored for the conditioned hallucinations task, and has been used in previous studies^10,13–15^.

To assess how the previously significant fixed effects related to HGF parameters, we refit the previously identified best-fitting GLMMs, replacing the behavioral outcomes with HGF-derived computational parameters as Gaussian outcomes. To address non-linearity in our fitted models, Box–Cox transformations were applied to HGF parameters that showed significant deviations from normality in model diagnostics.

### Exploratory analysis: evaluating non-linear trajectories in behavior and belief-weights

The goal of our first exploratory analysis was to further understand the nature of the time-dependent effects of spider phobia on conditioned hallucinations and hierarchical belief weights. By characterising the shape of these trajectories, we can test whether phobia-related effects reflect an altered starting point with linear decay versus a non-linear pattern indicative of dynamic changes in learning. To explore non-linear relationships over time, we implemented generalized additive mixed models (GAMMs) using the mgcv package^57^ to estimate a smooth term for time in its interaction with spider-phobia, while keeping the rest of the fixed and random effects the same. These models extend GLMMs by allowing for non-linear effects to be approximated through regression splines. To minimize overfitting, a penalty term is applied to the estimation of smooth terms, limiting excessive curve complexity. The flexibility of each smooth term is quantified by its effective degrees of freedom (EDF): an EDF of 1 indicates a linear effect, while values greater than 1 reflect increasingly non-linear relationships.

### Exploratory analysis: relating response patterns to changes in sensitivity and bias

The goal of our second exploratory analysis was to understand how the observed changes in response patterns relate to underlying shifts in perceptual sensitivity versus response bias within a signal detection theory (SDT) framework. This approach decomposes performance into two components: sensitivity (d′), reflecting the ability to discriminate signal from noise, and response criterion (c), reflecting a participant’s bias to favor one type of response over another, regardless of actual stimulus presence. In our task, no-tone trials served as the noise condition, while signal trials were defined by three levels of tone intensity estimated via the QUEST staircase procedure (25%, 50%, and 75%). We computed SDT measures separately at each intensity level, allowing us to assess how sensitivity and bias varied as a function of stimulus strength.

Using these derived parameters as Gaussian outcomes, we then refitted GAMMs using the same fixed and random effect structure as before. This left us with a total of six models, allowing us to model the time-varying trajectories of perceptual sensitivity (d′) and response criterion (c) separately for each of the three tone-intensity levels (25%, 50%, and 75%).

## Results

### Descriptive statistics

An overview of the descriptives for the final sample can be found in Table 1. The final sample for data analysis consisted of 447 participants (flowers = 229; spiders = 218). One participant had missing data for age, while seven participants had missing data for image ratings. The two groups did not significantly differ overall in terms of demographics, scores on self-report questionnaires, or their average ratings of spider and flower images (all *p* > 0.05, see Table 1). When comparing the overall ratings for the two classes of images (*n* = 440), participants rated spider images (as compared to flower images) as significantly more arousing (spiders: M = 4.99, SD = 2.76; flowers: M = 2.72, SD = 2.01; t(439) = 16.53, *p* < 0.001), more negatively valenced (spiders: M = -2.23, SD = 1.85; flowers: M = 1.72, SD = 1.65; t(439) = -33.13, *p* < 0.001), and less prototypical (spiders: M = 5.01, SD = 2.16; flowers: M = 6.30, SD = 1.60; t(439) = -11.40, *p* < 0.001). QUEST thresholding intensity estimates were not statistically different between the two groups (spiders: M = 60.00, SD = 2.89; flowers: M = 59.64, SD = 2.68; *t*(445) = -1.38, *p* = 0.169), and were not significantly correlated with hallucination-proneness (*r*(445) = 0.06, *p* = 0.225), trait anxiety (*r*(445) = − 0.09, *p* = 0.054), or spider-phobia (*r*(445) = 0.03, *p* = 0.471). In terms of correlations between predictors, hallucination proneness and trait anxiety were strongly correlated with each other (*r*(445) = 0.43, *p* < 0.001), while spider phobia was moderately correlated with trait anxiety (*r*(445) = 0.30, *p* < 0.001) and hallucination-proneness (*r*(445) = 0.28, *p* < 0.001). Additionally, spider phobia was strongly related to ratings of spider images in terms of arousal (*r*(438) = 0.52, *p* < 0.001) and valence (*r*(438) = − 0.56, *p* < 0.001) but not significantly related to prototypicality (*r*(438) = − 0.04, *p* = 0.397).

**Table 1:** Descriptive statistics.

Demographics	Flowers (*N* = 229)	Spiders (*N* = 218)	*p*-value	Total (*N* = 447)
Age^†^	33.0 (9.7)	34.4 (10.4)	0.151	33.7 (10.0)
Gender				
Male (%)	137 (59.8%)	130 (59.6%)	0.618	267 (59.7%)
Female (%)	91 (39.7%)	88 (40.4%)		179 (40.0%)
Non-binary/unspecified (%)	1 (0.4%)	0 (0%)		1 (0.2%)
Education Level				
Did not complete high-school	0 (0%)	1 (0.5%)	0.781	1 (0.2%)
High-school completed (%)	77 (33.6%)	72 (33.0%)		149 (33.3%)
Bachelor’s degree completed (%)	107 (46.7%)	101 (46.3%)		208 (46.5%)
Post-graduate degree completed (%)	45 (19.7%)	44 (20.2%)		89 (19.9%)
Self-Report Questionnaires				
STAI Score (20–80)	43.4 (12.1)	43.0 (12.6)	0.731	43.2 (12.4)
SDS Score (0 − 102)	51.2 (28.7)	49.9 (26.9)	0.615	50.5 (27.8)
LSHS Score (0–64)	16.9 (11.7)	17.7 (12.6)	0.491	17.3 (12.1)

Image ratings (avg)	Flowers (*N* = 226)	Spiders (*N* = 214)	*p*-value	Total (*N* = 440)
Spiders				
Valence (-5 – +5)	-2.30 (1.91)	-2.17 (1.79)	0.448	-2.23 (1.85)
Arousal (0–10)	4.99 (2.85)	4.98 (2.67)	0.965	4.99 (2.76)
Prototypicality (0–10)	4.98 (2.26)	5.04 (2.06)	0.766	5.01 (2.16)
Flowers				
Valence (-5 – +5)	1.58 (1.77)	1.87 (1.50)	0.065	1.72 (1.65)
Arousal (0–10)	2.83 (2.07)	2.61 (1.95)	0.255	2.72 (2.01)
Prototypicality (0–10)	6.26 (1.64)	6.35 (1.55)	0.527	6.30 (1.60)

Values are presented as mean (standard deviation) for continuous variables and as counts (percentages) for categorical variables. *p*-values for continuous variables are calculated using independent t-tests, while *p*-values for categorical variables are calculated using chi-square tests of independence. ^†^Age applies to 446 participants due to 1 missing data point.

### Behavioral outcomes: predicting conditioned hallucination frequency and confidence

To assess how threat relevance influenced conditioned hallucinations, we fitted GLMMs to predict perceptual decision-making on no-tone trials in terms of (1) the probability of reporting a “yes” response and (2) confidence ratings conditional on these “yes” responses.

Following hierarchical model comparison, we found the same optimal model structure for both the confidence and response models. For the random effects, hierarchical model comparison found that a model with random intercepts and slopes (over time) resulted in significantly better model fit over a model with random intercepts alone (response: χ²(2) = 411.65, *p* < 0.001; confidence: χ²(2) = 396.98, *p* < 0.001). For the fixed effects, both models retained three significant predictors: a three-way interaction between group, time, and spider phobia (response: χ²(1) = 3.85, *p* = 0.0498, β = −0.088, 95% CI [− 0.175, − 0.001]; confidence: χ²(1) = 4.81, *p* = 0.028, β = −0.092, 95% CI [− 0.174, − 0.010]), a main effect of hallucination-proneness (response: χ²(1) = 5.96, *p* = 0.015, β = 0.148, 95% CI [0.030, 0.265]; confidence: χ²(1) = 9.65, *p* = 0.002, β = 0.152, 95% CI [0.057, 0.248]), and a main effect of trait anxiety (response: χ²(1) = 4.33, *p* = 0.037, β = −0.126, 95% CI [− 0.243, − 0.008]; confidence: χ²(1) = 17.92, *p* < 0.001, β = −0.206, 95% CI [− 0.300, − 0.112]). No significant group differences were found when spider phobia was not considered, either in the main effect of group (response: χ²(1) = 0.44, *p* = 0.506, β = 0.071, 95% CI [-0.137, 0.279]; confidence: χ²(1) = 0.85, p = 0.357, β = -0.079, 95% CI [-0.247, 0.089]) or in the group x time interaction (response: χ²(1) = 0.08, *p* = 0.779, β = 0.011, 95% CI [-0.086, 0.108]; confidence: χ²(1) = 1.32, *p* = 0.251, β = -0.048, 95% CI [-0.131, 0.034]).

**Fig. 3:**
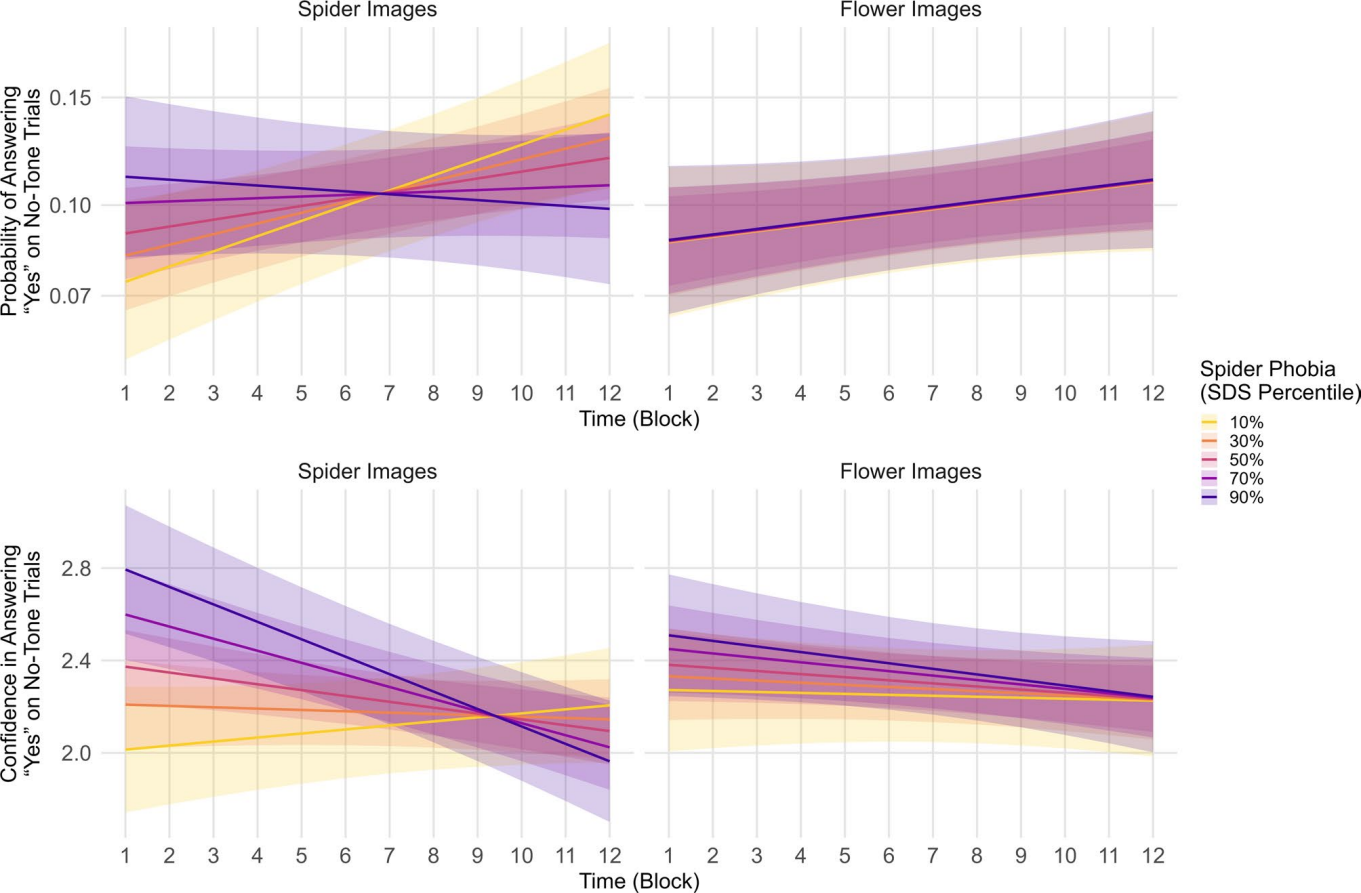
Plots showing the interaction effect of image condition and spider phobia on linear changes in response probability and confidence in conditioned hallucinations over time. Spider phobia levels are based on percentiles in SDS score; shaded areas show 95% confidence intervals.

To further investigate the nature of the three-way interaction effect, we fitted GLMMs separately for each group. A significant interaction between spider phobia and time was observed for the spider image group (response: χ²(1) = 6.76, *p* = 0.009, β = −0.086, 95% CI [− 0.150, -0.022]; confidence: χ²(1) = 14.29, *p* < 0.001, β = −0.077, 95% CI [− 0.117, − 0.038]), but not for the flower image group (response: χ²(1) < 0.01, *p* = 0.999, β ≈ 0.000, 95% CI [− 0.059, 0.059]; confidence: χ²(1) = 0.83, *p* = 0.361, β = −0.016, 95% CI [− 0.049, 0.018]). The plotted trajectories show that, for the spider images group, higher spider phobia is associated with more negative linear slopes in conditioned hallucinations over time (see Fig. 3): in the response model, positive slopes flatten, whereas in the confidence model, negative slopes steepen.

For the interpretation of main effects, participants with higher hallucination-proneness reported more frequent and confident hallucinations, while those with higher trait anxiety reported fewer and less confident hallucinations. The influence of hallucination-proneness and trait anxiety did not vary significantly by image group or over time (all *p* > 0.05; see Supplementary Material Tables S1 and S2).

### Computational outcomes: relating predictors to the weighting and use of prior beliefs

We next evaluated how the fixed effects identified in the behavioral model related to computational parameters, with a focus on the hypothesised role of hierarchical belief weights (X1, X2, X3) and reliance on prior beliefs (*v*). To do this, we extracted computational parameters from a hierarchical gaussian filter (HGF) based on our response data and refitted the previous final GLMMs with each HGF parameter entered as a Gaussian outcome. To address non-linearity in our fitted models, Box–Cox transformations were applied to HGF parameters, with the exception of the perceptual (X1) and contingency (X2) belief-weight models (see Supplementary Material Sect. 6.1).

In terms of estimated belief-weights at different hierarchical levels, the three-way interaction of group, time, and spider phobia significantly predicted changes in weightings at the contingency belief level (X2: χ²(1) = 6.93, *p* = 0.008, β = −0.098, 95% CI [− 0.171, − 0.025]), but not at the volatility belief level (Box–Cox transformed X3: χ²(1) = 0.11, *p* = 0.740, β = 0.001, 95% CI [− 0.007, 0.010]). For the perceptual belief level (X1), although an effect was observed with conventional standard errors (X1: χ²(1) = 4.10, *p* = 0.043, β = −0.009, 95% CI [− 0.018, − 0.000]), this effect was not robust to correction for heteroskedasticity (see Supplementary Material Sect. 6.1.1).

**Fig. 4:**
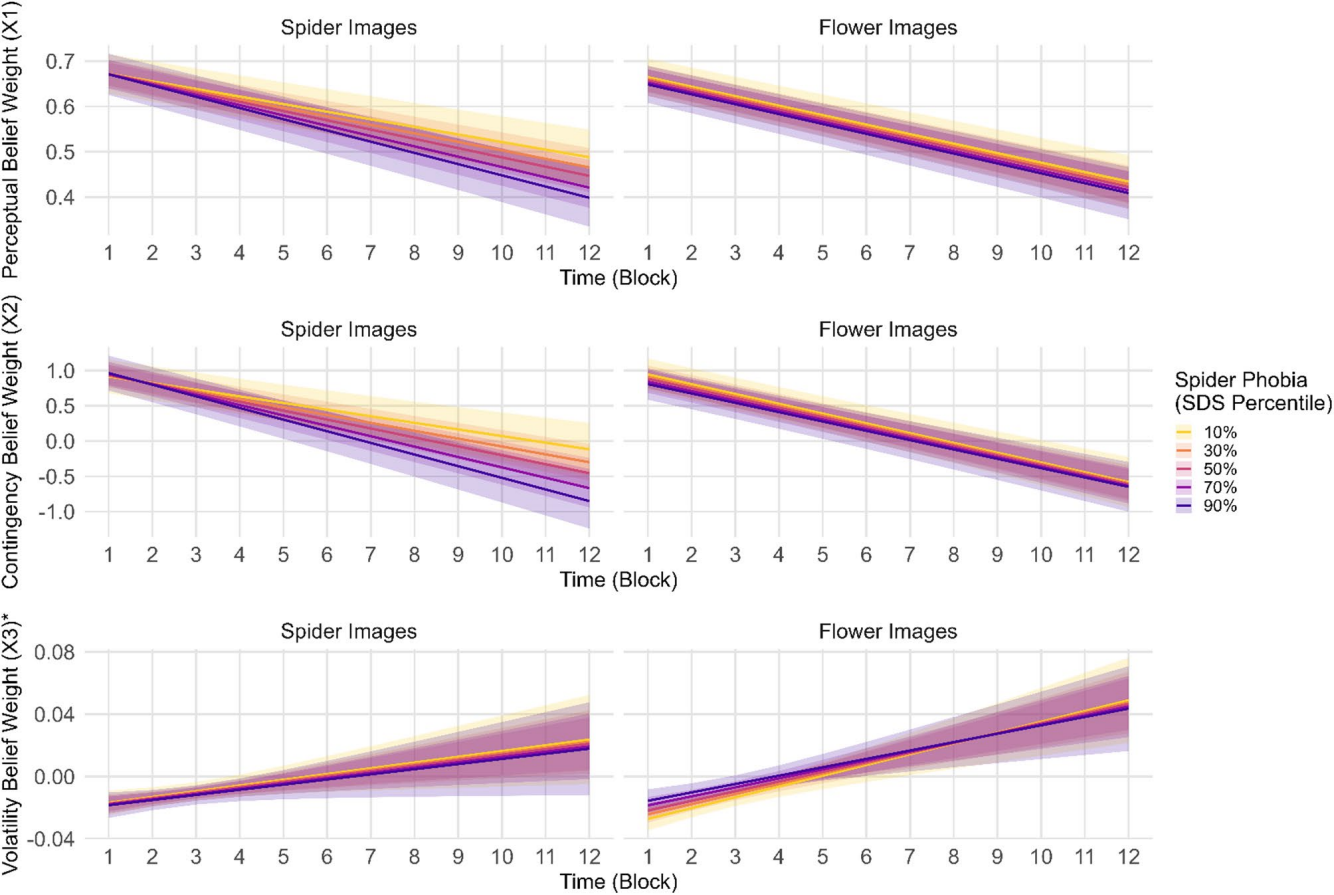
Model-predicted trajectories showing the interaction of spider phobia (SDS score) and time (experimental blocks) in predicting belief weights for spider (left) and flower (right) image groups. Lines represent predicted trajectories across five levels of spider phobia (10th to 90th SDS percentile), with shaded areas indicating 95% confidence intervals. *Note: Volatility belief-weights (X3) were Box-Cox transformed to address non-linearity in the originally fitted model.

Post-hoc models fitted within each group showed that individuals with higher spider phobia exhibited a sharper decline in the weighting of perceptual and contingency beliefs across the task when conditioned with spider images (X1: χ²(1) = 7.94, *p* = 0.005, β = −0.010, 95% CI [− 0.017, − 0.003]; X2: χ²(1) = 11.54, *p* < 0.001, β = −0.090, 95% CI [− 0.141, − 0.038]; see Fig. 4). In contrast, when conditioned with flower images, no significant interaction effects were observed for spider phobia over time at either level (X1: χ²(1) = 0.15, *p* = 0.696, β = −0.001, 95% CI [− 0.006, 0.004]; X2: χ²(1) = 0.10, *p* = 0.753, β = 0.008, 95% CI [− 0.043, 0.060]).

Additionally, hallucination-proneness was significantly related to increased weightings at both the perceptual (X1: χ²(1) = 5.24, *p* = 0.022, β = 0.022, 95% CI [0.003, 0.041]) and contingency (X2: χ²(1) = 6.29, *p* = 0.012, β = 0.136, 95% CI [0.030, 0.242]) levels of belief, but not at the volatility level (Box–Cox transformed X3: χ²(1) = 1.55, *p* = 0.212, β = −0.002, 95% CI [− 0.004, 0.001]). No significant relationships were observed for trait anxiety across any of the belief levels (X1: χ²(1) = 0.51, *p* = 0.474, β = −0.007, 95% CI [− 0.026, 0.012]; X2: χ²(1) = 1.40, *p* = 0.236, β = −0.064, 95% CI [− 0.171, 0.043]; Box–Cox transformed X3: χ²(1) = 1.94, *p* = 0.164, β = 0.002, 95% CI [− 0.001, 0.005]).

We evaluated how our fixed effects related to the HGF estimate of participants’ reliance on prior beliefs versus sensory evidence during the task (Box–Cox transformed *v*). We found no interaction effect of group and spider phobia (χ²(1) = 0.64, *p* = 0.423, β = 0.071, 95% CI [− 0.104, 0.245]), nor an overall effect of group when differences in spider phobia were ignored (χ²(1) = 3.15, *p* = 0.076, β = 0.156, 95% CI [− 0.017, 0.329]). Independent of group, we observed main effects of hallucination-proneness (χ²(1) = 6.41, *p* = 0.011, β = 0.125, 95% CI [0.028, 0.223]) and trait anxiety (χ²(1) = 5.65, *p* = 0.017, β = −0.118, 95% CI [− 0.216, − 0.020]), indicating that hallucination-prone individuals showed a relative overreliance on prior beliefs, whereas trait-anxious individuals showed a relative overreliance on sensory evidence in formulating perceptual decisions. We explored whether our predictors were significantly related to other extracted parameters from the HGF and found no further significant relationships, either for decision noise (ζ) and for evolution rates encoding transition probabilities (ω₂, ω₃, all *p* > 0.05, see Supplementary Material Sect. 6.1.2).

### Exploratory analysis: evaluating non-linear trajectories in behavior and belief-weights

To further investigate the nature of the time-dependent effects of individual threat-relevance, we explored non-linear trajectories in behavior and belief-weights across the task. To do this, we used GAMMs to estimate a smooth term for time in its interaction with spider-phobia, while keeping the rest of the fixed and random effects the same. We also evaluated whether these trajectories were specific to conditioned hallucinations (i.e. decision-making on no-tone trials) or whether they reflected decision making for hearing tones more generally (i.e. decision-making on all trials). This exploratory analysis focuses on participants conditioned with spider images where group-specific time-dependent variations were observed (see Supplementary Material Sect. 7.1 and 8.1 for flower group results based on the same analyses).

We found evidence for significant non-linear interaction effects for all of our behavioral and computational outcomes (all edf > 3, *p* < 0.05) except for confidence in conditioned hallucinations, which showed a significant but linear interaction (edf = 1.00, F = 14.72, *p* < 0.001). Specifically, spider phobia was significantly related to non-linear changes over time for: (1) the probability of reporting tones on no-tone trials (edf = 3.33, F = 4.65, *p* = 0.012) and across all trials (edf = 3.49, F = 10.96, *p* < 0.001); (2) confidence in hearing tones across all trials (edf = 3.44, F = 8.42, *p* < 0.001); and (3) perceptual and contingency belief-weights extracted from the HGF (X1: edf = 3.11, F = 7.24, *p* < 0.001; X2: edf = 3.10, F = 10.73, *p* < 0.001).

**Fig. 5:**
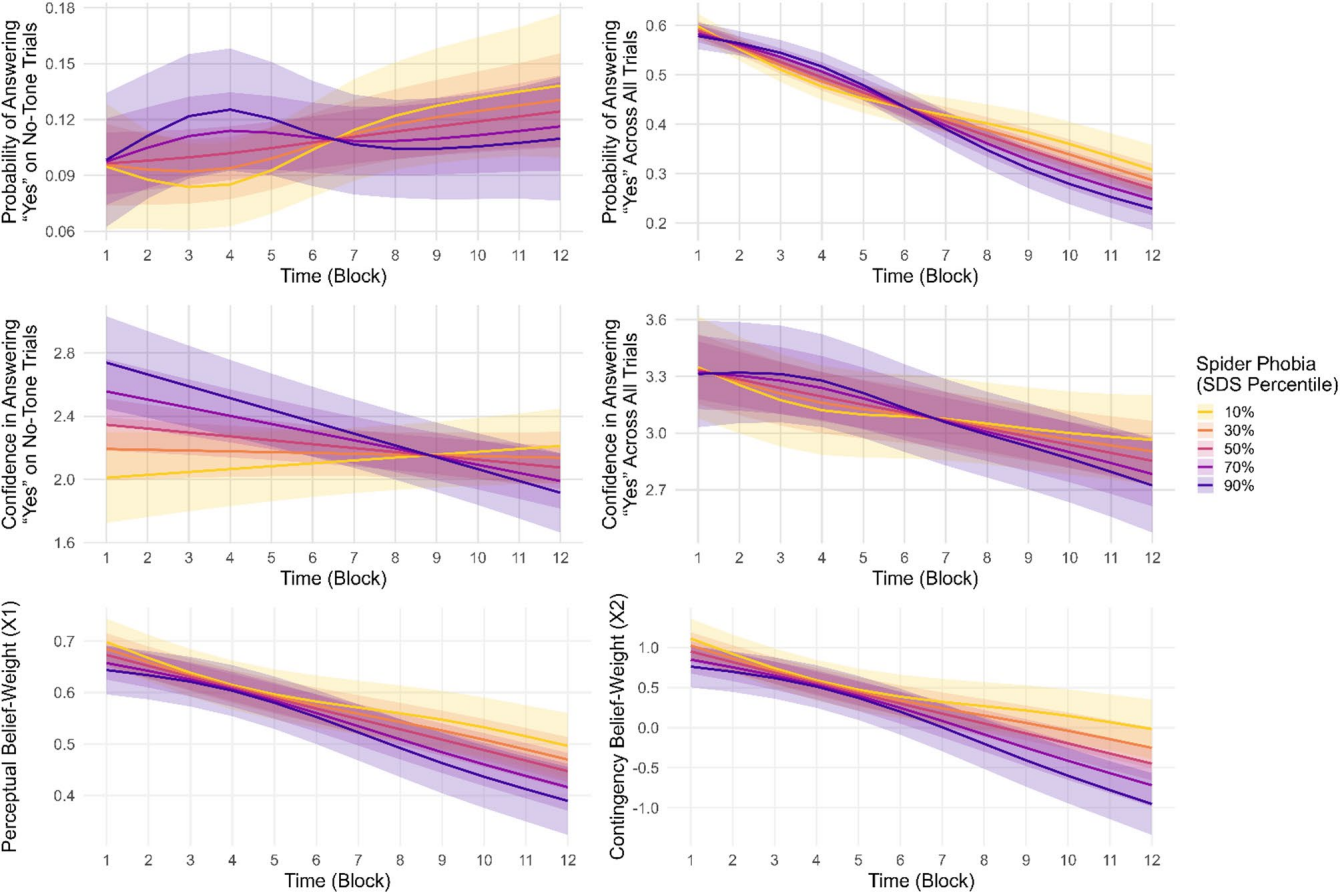
Model-predicted trajectories from GAMMs estimating a smooth term for time in its interaction with spider phobia across six key outcomes. The top row shows the probability of reporting hearing a tone on no-tone trials (left) and across all trials (right). The middle row shows confidence in reporting tones on no-tone trials (left) and across all trials (right). The bottom row depicts perceptual (X1; left) and contingency belief-weights (X2; right). Lines represent predictions for five levels of spider phobia (10th to 90th percentile of SDS score); shaded areas indicate 95% confidence intervals.

The fitted non-linear trajectories can be found in Fig. 5. The plot suggests that spider phobia is related to an increase in reporting conditioned hallucinations in early to middle phases, followed by a reversal and eventual decrease in reporting conditioned hallucinations in later phases. Behavioral changes in the probability of and confidence in reporting tones show a similar trajectory but tend to emphasise changes in later phases where high spider phobia is related to less frequent and confident reports of hearing tones. Changes in belief-weighting, which are extracted based on response patterns across all stimulus levels, also emphasise non-linear changes in later phases leading to weaker perceptual and contingency beliefs.

### Exploratory analysis: relating response patterns to changes in sensitivity and response bias

We finally explored how behavioral changes could be decomposed into SDT parameters, dissociating changes in stimulus discrimination sensitivity (*d*′) from changes in response bias (*c*) at the different QUEST stimulus intensity levels (25%, 50%, 75%).

**Fig. 6:**
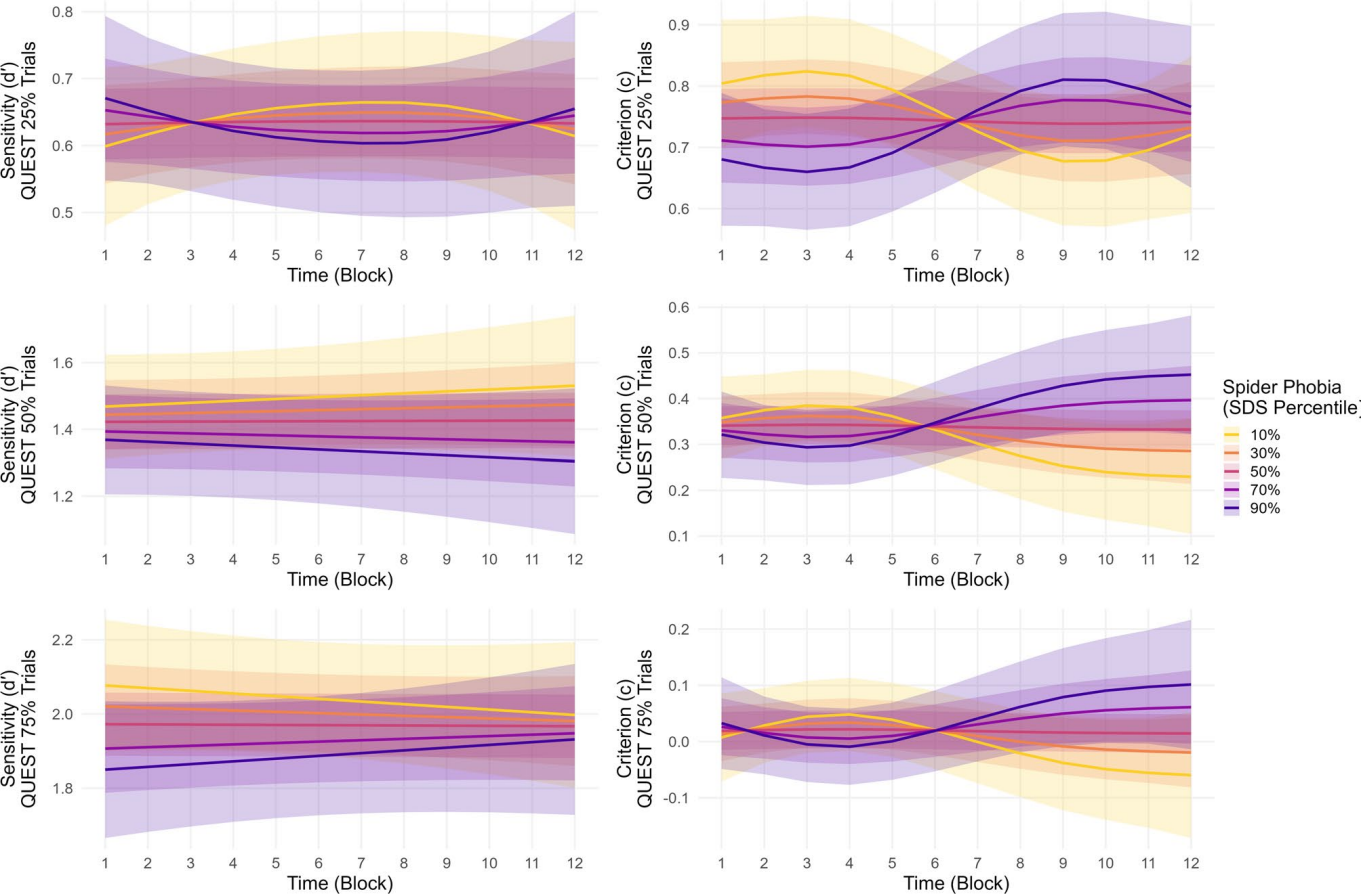
Predicted trajectories of sensitivity (d′, left) and criterion (c, right) over time, separated by QUEST difficulty levels: 25% (top), 50% (middle), and 75% (bottom). Lines represent predictions for five levels of spider phobia (10th to 90th percentile of SDS score); shaded areas show 95% confidence intervals.

We observed that, when conditioned with spider images, increased spider phobia was significantly related to non-linear changes in response bias (c) over time at 25% (edf = 3.02, F = 4.78, *p* = 0.002) and 50% (edf = 2.79, F = 5.07, *p* = 0.004) stimulus intensity levels. A similar non-linear pattern was observed at 75% stimulus intensity, although this did not reach statistical significance (edf = 2.75, F = 3.39, *p* = 0.054). On the other hand, spider phobia was not significantly related to linear or non-linear changes in sensitivity (*d*′) over time at any stimulus intensity level (25%: edf = 1.90, F = 0.95, *p* = 0.353; 50%: edf = 1.00, F = 0.38, *p* = 0.536; *75%: *edf = 1.00, *F* = 0.54, *p* = 0.462). Notably, response bias trajectories mirrored the general shape of trajectories observed for behavior, as higher spider phobia was related to a more liberal response criterion in early to middle phases followed by a more conservative response criterion in later phases (see Fig. 6). Similar to the differences in behavior observed in ‘no-tone’ trials and across all trials, response criterion shifts for low stimulus-intensity trials (QUEST 25%) emphasized changes in early phases, while high stimulus-intensity trials emphasized changes in late phases (QUEST 75%), with medium stimulus-intensity trials falling somewhere in between (QUEST 50%).

## Discussion

In this study, we investigated the influence of threat-relevance on conditioned hallucinations and the moderating roles of individual differences in spider phobia, trait anxiety, and hallucination proneness. Drawing on theories linking threat-related learning to rigid priors and hallucinations in psychopathology^6,7,44^, we employed a HGF model to examine how these effects related to how participants weighted their prior beliefs during the task. Contrary to a simple, directional relationship implied by our preregistered hypotheses, we found a nuanced time-dependent effect of individual threat-relevance on conditioned hallucinations, which was linked to increased belief updating during the task. Additionally, we found that hallucination-proneness and trait anxiety, despite being positively correlated with one another, are inversely related to conditioned hallucinations and the weighting of prior beliefs. Taken together, these findings suggest that threat-relevance sensitises beliefs underlying conditioned hallucinations, challenging the view that threat-related learning promotes rigid priors in clinical hallucinations.

In our preregistration, we hypothesised that threat-relevance could either (1) increase conditioned hallucinations, reflecting the theorized role of threat-related learning in psychopathological hallucinations, or (2) reduce conditioned hallucinations by impairing associative learning due to a distraction effect. We found negative or ambiguous support for these preregistered hypotheses. Specifically, threat-relevance had no significant effect on overall conditioned hallucination rates, neither by group (spider images vs. flower images) nor through its interaction with spider phobia. Furthermore, we found no significant influence of threat on the relative use of prior beliefs (*v*) during the task. However, we did observe a significant time-dependent effect of individual threat-relevance: participants in the spider image group with higher spider phobia appeared to show an increase in conditioned hallucinations during early phases, followed by a relative drop in conditioned hallucinations during later phases. These changes were linked to accelerated belief-updating in the HGF model and, according to a SDT framework, shifts in participants’ internal threshold for deciding whether or not a tone was present. While this pattern points to an influence of individual threat-relevance, it falls outside the scope of our original hypotheses, which predicted global increases or decreases in hallucination rates but did not specify how threat might dynamically influence conditioned hallucinations at different experimental time-points.

One way to more precisely relate the present findings to our original hypotheses is to derive more specific predictions concerning how threat-relevance impacts conditioned hallucinations in relation to changing stimulus contingencies during the task. The hypothesis that threat facilitates hallucinations via biased associative learning derives from literature proposing that threat-relevant priors are more rapidly acquired^44^ and more difficult to unlearn^6,7,44^. This aligns with our early-phase finding of increased conditioned hallucination frequency and confidence for higher levels of spider-phobia (indicating more rapid prior acquisition) but conflicts with later-phase drops in conditioned hallucinations and accelerated belief updating leading to weaker perceptual and contingency beliefs (suggesting rapid extinction). In contrast, the distraction hypothesis predicts impaired associative learning and insensitivity to contingency relationship changes, which fits the pattern of weaker beliefs in late phases but contradicts both early increased susceptibility to hallucinations and the overall trend of rapid belief-updating during the task. Moreover, a distraction effect would likely produce noisier decision-making, which was not supported by either the HGF response model (no change in ζ) or the SDT framework (no change in d’). Thus, even with more targeted, time-sensitive predictions, our behavioral data do not clearly support either preregistered hypothesis.

We believe our results could point to a third possibility: an ‘accelerated belief-updating’ model where threat-relevance aligns perception more closely with recent stimulus contingencies, increasing both the rate of learning (acquisition) and unlearning (extinction) of stimulus associations. This would explain both the increase in the frequency and confidence of conditioned hallucinations in early to middle phases (during which spiders more reliably predicted tones) and the marked reduction in conditioned hallucinations in later phases accompanied by accelerated belief-weight decay (as these predictive associations weakened). Furthermore, it is consistent with the observed changes in criterion from early to late phases, as participants more flexibly adapt their internal decision criterion to changing external stimulus contingencies during the task. Importantly, similar patterns were observed for both response probability and confidence, and computational modelling linked these effects to changes in prior belief-weighting rather than response parameters, arguing against a purely decisional account. While this alternative explanation provides a potential account of the observed time-dependent effects, it is post-hoc and thus requires further work to evaluate its empirical validity. Future studies could test this model by reversing the stimulus contingencies in the conditioned hallucinations task design, presenting weak contingencies first and strong contingencies later, which should produce opposite behavioral trends.

Mechanistically, accelerated belief-updating could be driven by a tendency of spider-phobic individuals to allocate increased attentional resources to spider-related cues during the task^58^. This could enhance the relative processing of stimulus contingencies based on the relationship between the conditioning stimulus (spider images) and the target (neutral tone). Alternatively, accelerated belief-updating could be driven by increased physiological arousal in spider-phobic individuals^59^, leading to a state which is sensitized to changing stimulus contingencies, independent of stimulus content. Future studies incorporating stress-manipulations and more precise physiological measures of arousal (e.g., pupil dilation, skin conductance) could help shed further light on the potential contribution of these different influences^61^. Furthermore, physiological measures, or repeated image ratings of arousal and valence across the task, could help determine how fluctuations in affective state (e.g., due to emotional habituation) relate to underlying changes in perceptual decision-making processes.

The notion that threat-relevance accelerates belief-updating appears to conflict with the theorized role of threat-related learning in the development of overly rigid priors^44^. For example, the prior beliefs underlying psychopathological hallucinations in PTSD are characteristically resistant to updating in the face of conflicting evidence^6,7,45^, while the prior beliefs underlying conditioned hallucinations in this task were more flexibly unlearnt as stimulus contingencies were weakened. We highlight two task-design considerations that could contribute to this inconsistency.

The first consideration lies in whether threat functions as a predictive cue for hallucinations or as an intrinsic part of the hallucination itself. The hallucinations in psychopathology frequently include negatively valenced and threatening contents (e.g., persecutory voices^33^), whereas the content of the stimulus-evoked hallucinations in this study (sine-wave auditory tones) are assumed to be affectively neutral prior to conditioning. While our study evaluates how threat-related learning impacts conditioned hallucinations, a closer approximation to clinical hallucinations would involve a task design where participants learn to predict the presence or absence of a threatening stimulus (e.g., a spider image) based on a neutral cue (e.g., an auditory tone). This better reflects hallucinations driven by learnt ontogenetic threats, such as in PTSD, where previously neutral contexts can trigger trauma-related hallucinations^6^. We initially attempted this exact design (see previous versions of our preregistration 10.17605/OSF.IO/P3XG8); however, we were unable to engender a conditioning effect (even in the control group) and staircasing multiple images simultaneously was time-consuming and unreliable. This distinction may be important for understanding the apparent differences in learning dynamics during extinction: unlike in real life, where extinction involves neutral contexts that no longer predict threat, in this study, the threatening stimulus remained constant during extinction, and only its association with the neutral cue was weakened. From this perspective, acquired threat-related associations may be either flexibly or inflexibly unlearnt depending on the presence of threat during the extinction process. Future paradigms that can reliably induce threat-related hallucinations based upon neutral cues could help clarify this influence.

A second potential contributing factor is the nature and intensity of the threat manipulation. Although participants in this study rated spider images as negatively valenced and emotionally arousing, the distress induced by passively viewing such images is negligible compared to the highly distressing and sometimes life-threatening experiences that underlie clinical hallucinations. The level of distress may be crucial towards understanding the influence of threat on the flexibility of prior beliefs: while mild stressors typically enhance associative learning, traumatic or highly stressful experiences impair it and are related to more inflexible behavioral strategies^60^. Spiders may serve as a convenient methodological proxy for threat; however, their impact may not generalize to real-world threats involved in psychopathology.

Although our primary findings relate to the role of threat-relevance, there are three further aspects of our study that we deem relevant for future research investigating conditioned hallucinations.

First, this study provides a novel finding concerning how hallucination-proneness and trait anxiety relate to one another in predicting conditioned hallucinations. In line with previous studies, we found that hallucination-prone individuals are more susceptible to conditioned hallucinations, weight perceptual and contingency beliefs more strongly (X1, X2), and rely more on prior beliefs (higher *v*) during perceptual decision-making^10,13–15^. We additionally show that trait anxiety tends to show an opposite effect, where participants experienced less frequent and confident conditioned hallucinations and rely less on prior beliefs in formulating perceptual decisions. This finding runs counter to another study showing that trait anxiety is related to an increased weighting of prior beliefs^62^. The inverse relationship of hallucination-proneness and trait anxiety is interesting, since the two are themselves strongly positively correlated (our results: *r* = 0.43) and both are implicated in psychopathology^63^. The results presented here suggest that the two may be associated with distinct alterations in perceptual decision-making processes. Future studies could examine whether modality-specific hallucinations and the presence of negative content (e.g., threatening voices) differentially predict susceptibility to threat-related conditioned hallucinations.

Second, the finding that trait anxious participants reported less confident and frequent conditioned hallucinations could be interpreted to run counter to the notion that hallucinations emerge as harm-avoidance response to perceived threat^44,46^. However, although trait anxiety is conceptually related to threat evaluation, meta-analytic findings show it is largely distinct from trait fear^64^: the two show only a moderate correlation across studies (*r* = 0.32) and this is even weaker when fear is conceptualized in terms of harm avoidance (*r* = 0.14). Future studies incorporating additional measures of trait fear can help clarify how the two relate to one another in predicting hallucination-related perceptual decision-making behaviors.

Third, this is the first study (to our knowledge) to demonstrate that conditioned hallucinations can be successfully elicited via multiple exemplars of a perceptual category. While our estimates are slightly lower than other studies using online samples^14^, these studies also conducted auditory-tone thresholding in the presence of visual stimuli, which may contribute to an increased conditioning effect during the task. Follow-up studies can evaluate whether the strength of conditioned hallucinations elicited by multiple stimuli varies as a function of (1) the number of conditioned items, and (2) the degree of low-level (contrast, luminance) and high-level (category) similarity of the items.

## Conclusion

The results of the present study suggest that the influence of threat-relevance on conditioned hallucinations is nuanced, with individual differences in threat-relevance playing an important moderating role. Contrary to the hypothesised role of threat-relevance in the development of rigid priors, we found evidence that threat-relevance is rather linked to flexible priors that are more rapidly updated in relation to changing stimulus contingencies. This study underscores the importance of considering time-dependent variations in conditioned hallucinations across both acquisition and extinction phases. A key challenge for future research will be to clarify the conditions under which threat promotes or suppresses the extinction of learnt associations underlying hallucinations. Belief-updating during extinction is of particular relevance to trauma-based hallucinations, such as in PTSD^6,7,45^, where deficits in extinction learning are found^65^. Important questions for future research concern the extent to which these results depend on the content of the conditioned hallucination (threatening vs. neutral), and the intensity of the threat-manipulation used. Addressing this gap could help isolate the causal factors underlying the rigidity of priors in psychopathology and inform more effective interventions for hallucinations that emerge in response to learnt threats.

## Supplementary Information

Below is the link to the electronic supplementary material.

Supplementary Material 1

## Data Availability

The research data and underlying code supporting the results and figures of the manuscript is hosted in the following OSF repository: https://doi.org/10.17605/OSF.IO/SRHQD.
