# Supplementary material for: Individual threat-relevance accelerates belief-updating in conditioned hallucinations
Source: Sci Rep. 2026 Jun 13;16:19557. doi: 10.1038/s41598-026-52299-9 (PMC13294484; doi:10.1038/s41598-026-52299-9)
Supplement: Supplementary file 1 — Supplementary Material 1 [file 41598_2026_52299_MOESM1_ESM.docx]

**Supplementary Material**

**1. Participant Exclusion Details**

We excluded 102 participants from our initial sample of 549 participants. Ninety of these exclusions were based on preregistered exclusion criteria, while a further 12 exclusions were justified based on non-preregistered reasons. We describe each of these in turn.

**1.1 Preregistered Exclusions**

The largest factor underlying participant exclusion in our study was based on preregistered metrics of poor task adherence. We had two main criteria based on performance during the conditioned hallucinations task [1]. First, we considered performance unacceptable when participants were more likely overall to report hearing softer tones than louder tones during the experiment. This was tested by estimating a linear slope for each participant’s response probability across the 25%, 50% and 75% QUEST intensity levels. Thirteen participants exhibited a negative linear slope for this relationship. Second, we deemed performance unacceptable if participants were increasingly likely to report hearing tones across blocks, given the higher proportion of louder tones in earlier blocks; sixty-three participants met this criterion, exhibiting a positive slope that reflected an increased propensity to report hearing tones in later blocks, despite them being, on average, softer. Based on this, a total of 65 participants were removed due to either a negative slope according to QUEST intensity level (n = 2), a positive slope for response probability across the experimental blocks (n = 52) or both (n = 11). A further 23 people were excluded due to preregistered criteria based on their answers to self-report questions. Specifically, participants were excluded if they reported a history of a cognitive (n = 18), neurological (n = 3), or seizure disorder (n = 3), or if they reported being under the influence of drugs or alcohol at the time of testing (n = 1). Because two participants reported two co-occurring conditions (cognitive + neurological; seizure + cognitive), the total number of exclusionary cases reported (25) is larger than the number of participants excluded (23). Finally, two further participants were excluded for being over the explicit cut-off age of 65.

**1.2 Further Non-Preregistered Exclusions**

In addition to the above, a further 12 participants were excluded based on reasons that were not considered in the preregistration. The first concerns the data frames of five participants, who did not compile/export correctly, leading to missing essential information of the stimulus intensity that was associated with each trial. Since this could not be reconstructed, these participants were excluded. The second concerns missing questionnaire data from a further four participants. Although this was not a specific preregistered criterion, the nature of our top-down modelling meant that all analyses required scores from self-report scales to be included. Thus, they were excluded. One participant had one missing LSHS item. Because the rest of their data was usable, they were included in the analysis. Their total LSHS score was estimated by calculating the mean of their completed items and scaling to the full number of items. Finally, three additional participants were removed due to suspicious data. In the original sample, four participants gave the response *“Spain”* to the question *“What year were you born in?”*. One of these four had already been excluded under the preregistered criteria described above, leaving three participants who remained in the dataset. The specificity and consistency of this clearly incorrect response led us to conclude that their data were at best unreliable and at worst fraudulent (e.g., automated responses), and so these three participants were removed.

**2. Image Selection Procedure**

We received an original sample of 29 images (15 flowers, 14 spiders) for use in our study. This image set has been previously used in a study evaluating the neural basis of affective salience [2]. To obtain the final set, we selected a balanced subset of 14 images (7 per group) that minimized differences in low-level physical variability between the two image categories. This was important because physical variability in a stimulus set (specifically the low-level similarity or dissimilarity of images within a category) could confound the effects of conditioning in our experiment. If one category (e.g., spiders) is more internally consistent in its physical characteristics than another (e.g., flowers), any group difference observed could potentially be attributed to those differences in similarity rather than to the hypothesized variable of interest, namely threat-relevance.

To control for this, we focused on the spatial frequency distributions of our image sets. For each image, we calculated the mean and standard deviation of its spatial frequency spectrum, and then compared the spread of these values across groups. In the full sample, the standard deviation of image means was substantially higher for flowers (SD = 5.80) than for spiders (SD = 3.32), yielding a difference of 2.48 units. Similarly, the standard deviation of image SDs was greater for flowers (SD = 4.43) compared to spiders (SD = 2.00), with a difference of 2.43 units. This suggested that the flower images were more physically variable, when analysed in terms of these low-level features.

Using an iterative image removal process, we created an optimized sample of 14 images (7 per group) that minimized these group differences. In the final set, the difference in standard deviation of image means was greatly minimized, with flowers now showing slightly lower variability (SD = 1.04) than spiders (SD = 1.31), a difference of -0.27 units. Similarly, the standard deviation of image SDs was reduced, with flowers (SD = 2.21) being slightly less variable than spiders (SD = 2.52), a difference of -0.31.

Importantly, spatial frequency is only one form of physical variability, and other perceptual or semantic differences may still influence participant responses. Nevertheless, this image selection process helped us minimize one source of low-level variability that could have potentially impacted our stimuli in the task.

**3. Experimental Stimuli and Description of Image Rating Scales**

***3.1 Examples of Experimental Images***


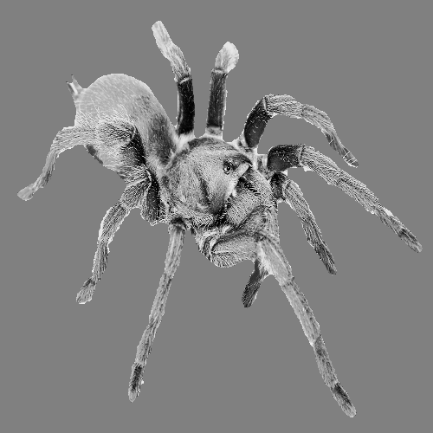

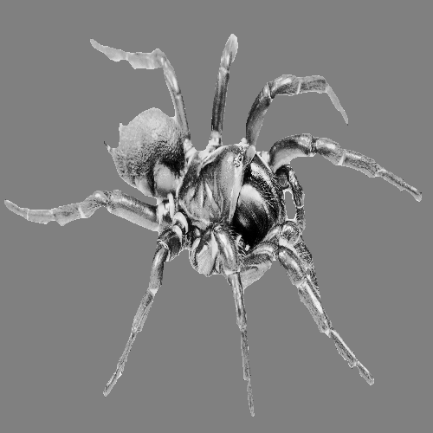

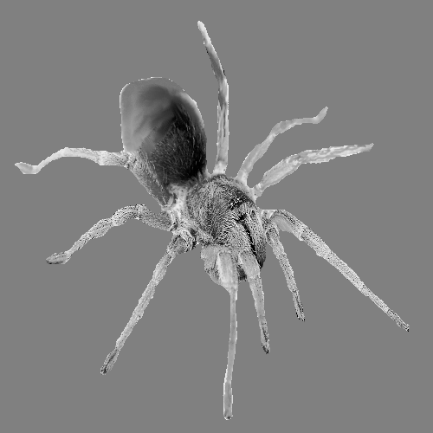


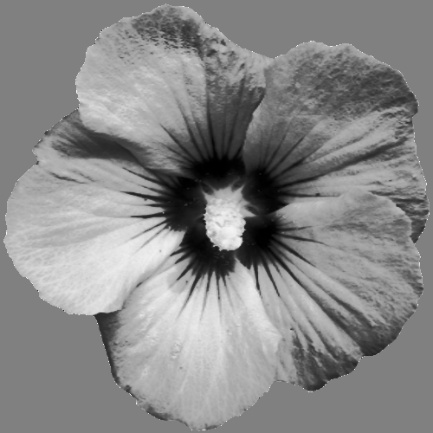

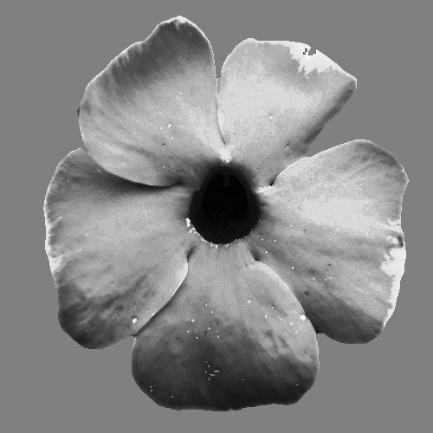

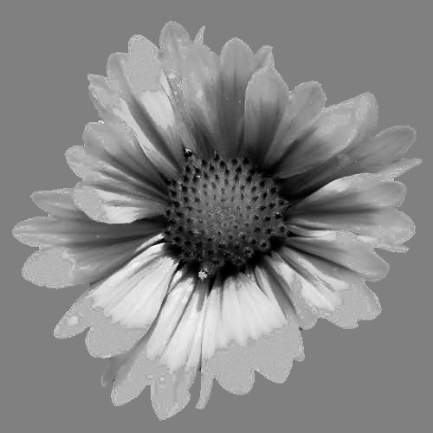


***3.2 Description of Image Rating Scales***

For image ratings, participants were shown all 14 experimental images and asked for each image to rate the image in terms of arousal, valence and prototypicality on a visual-analog scale.

For the valence scale, possible scores ranged from -5 to +5 where -5 indicates feeling very unpleasant; a score of +5 indicates feeling very pleasant, and a score of 0 indicates feeling neutral.

For the arousal scale, possible scores ranged from 0 to +10 where 0 indicates feeling very calm; +10 indicates feeling very aroused, and a score of +5 indicates feeling somewhat aroused.

For the prototypicality scale, possible scores ranged from 0 to +10 where 0 indicates the image is very atypical; +10 indicates that the image is very typical, and a score of +5 indicates that the image is somewhat typical.

**4.** **Additional Supplementary Plots**

***4.1 Correlations among self-report measures and image ratings***

**
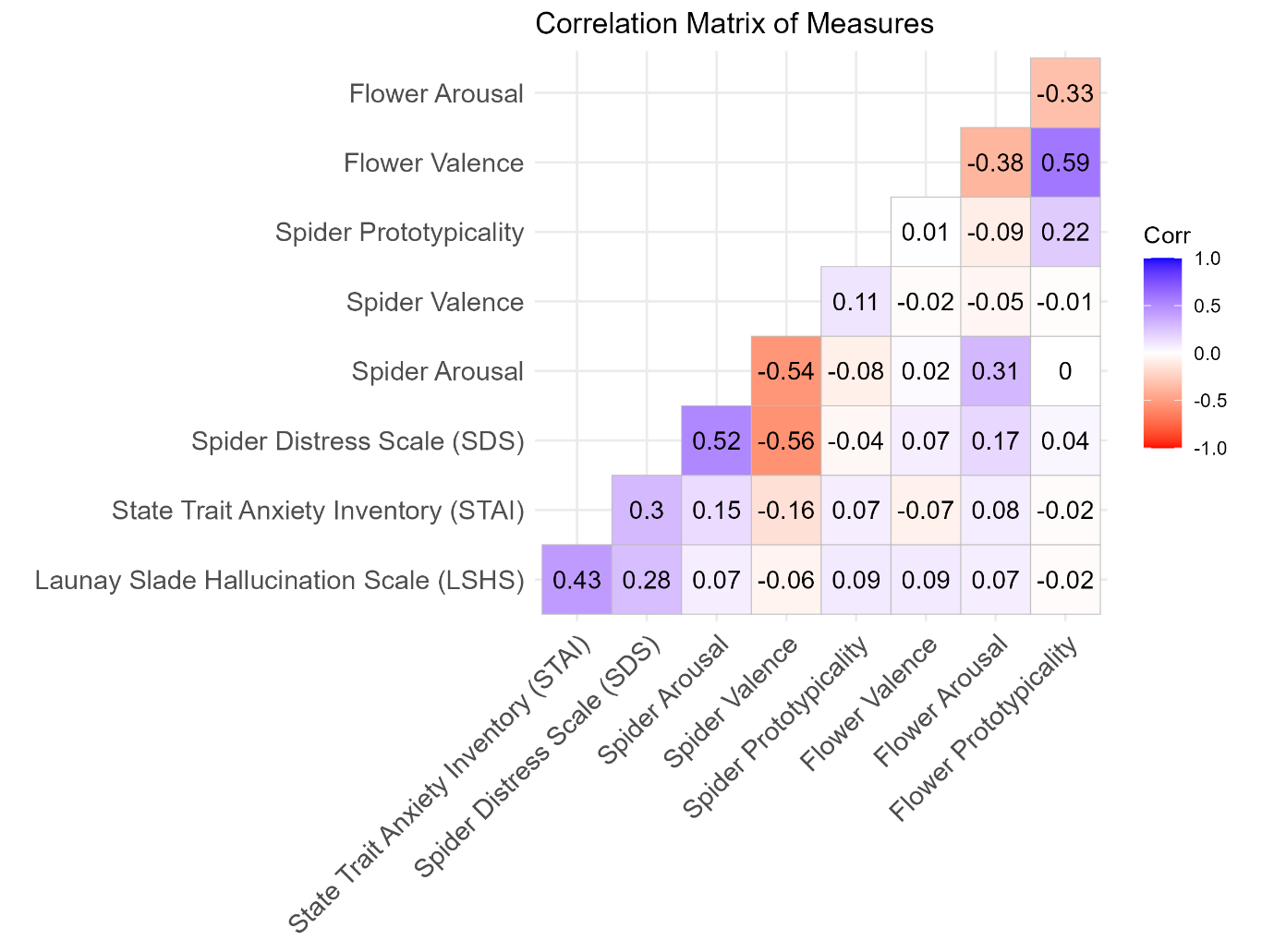
**

**Figure S1.** Pearson correlation matrix of participant-level scores across psychological scales (LSHS, STAI, SDS) and affective ratings (valence, arousal, and prototypicality) for spider and flower images.


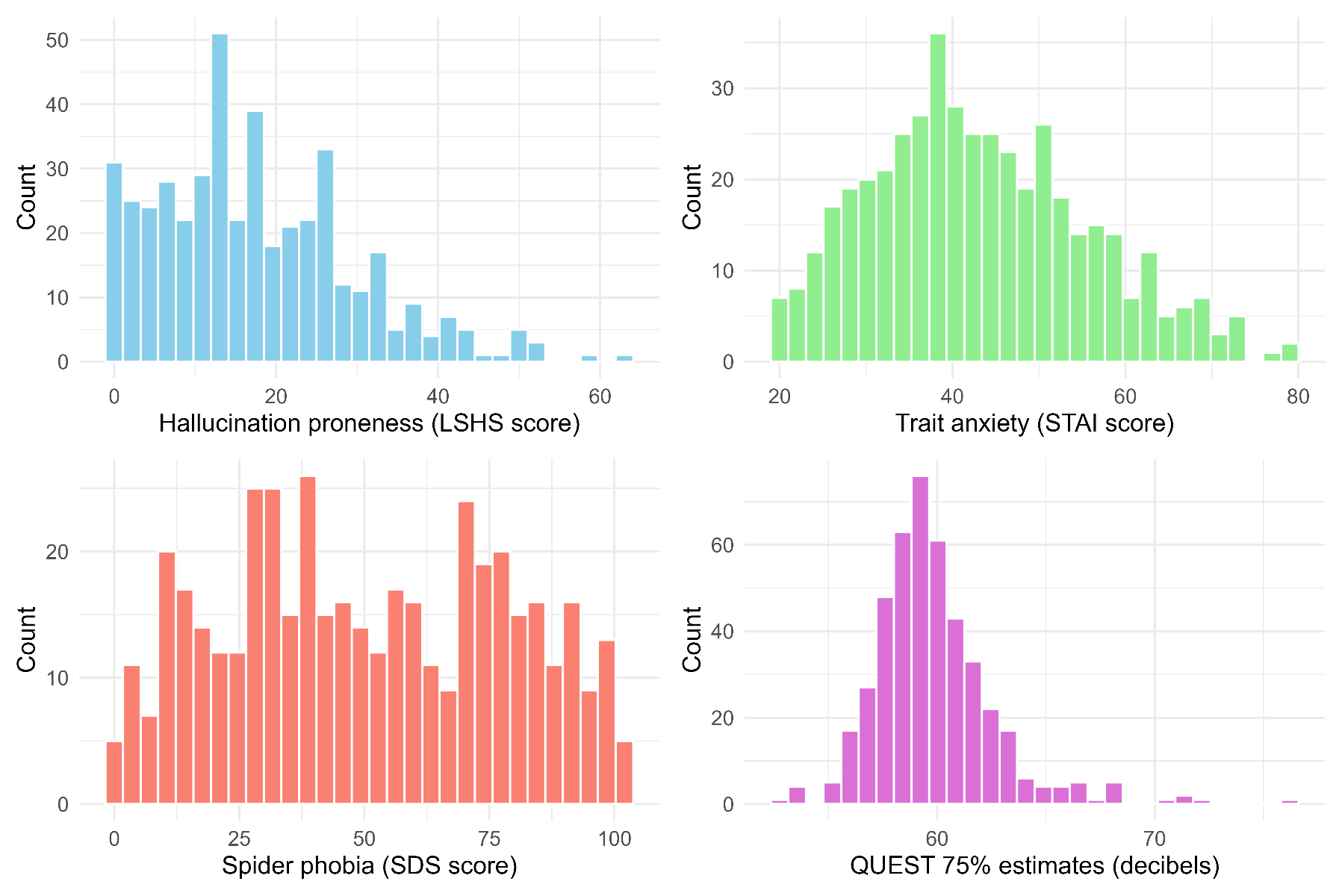


**Figure S2.** Distribution of psychometric scales and QUEST intensity estimates. Histograms show the distributions of scores on the (A) Launay–Slade Hallucination Scale Extended (LSHS), (B) Trait version of State–Trait Anxiety Inventory (STAI), (C) Spider Distress Scale (SDS), and (D) QUEST 75% threshold estimates.

**5. Behavioral Outcomes: Predicting Conditioned Hallucination Frequency and Confidence**

**5.1 Hierarchical model selection details**

To evaluate the contribution of fixed effects, we used a top-down method based on an approach by Zuur et al. [3]. We start with an ‘overly saturated model’ containing all of the possible fixed effects for our analysis, and then we iteratively remove non-significant effects until we arrive at a suitable model. Our original model consisted of three three-way interactions (including their lower-order terms).

Outcome ~ sds * block * group + lshs * block * group + stai * block * group

This initial model structure was justified based on a balance between model convergence and possible hypothesised effects. Interaction effects with self-report scales were only considered in relation to group and block, and not to one another, since four-way interactions become exponentially complex to estimate.

Variables were iteratively removed according to (1) their complexity and (2) their significance according to a log-likelihood ratio test. We started by evaluating the contribution of 3-way interactions, then we moved on to the 2-way interactions, and finally we evaluated the main effects. At each level, we compare how the removal of a fixed effect impacts the overall model fit according to a log-likelihood ratio test. A fixed effect is removed if it (1) has the lowest chi-square value at that level of complexity and (2) its removal does not significantly worsen the model fit (p > 0.05). Fixed effects are removed one at a time to avoid confounding effects between variables. A fixed effect is retained in the model if (1) its removal results in a significant difference in model fit based on a log-likelihood ratio test (p < 0.05) or (2) it constitutes a lower-order term of an existing significant interaction effect. This procedure is performed in a stepwise fashion until we arrive at a final model. Tables S1 and S2 below shows the step-by-step procedure of removing/retaining fixed effects to arrive at the final models for confidence in conditioned hallucinations and response patterns relating to conditioned hallucination reports.

**Table S1: Stepwise fixed effects selection process for the response model**

| Variable removed | AIC | χ² | *p*-value |
| --- | --- | --- | --- |
| None (full model) | 46980.53 | NA | NA |
| group:block:stai | 46978.55 | 0.018 | 0.892 |
| block:stai | 46976.60 | 0.056 | 0.813 |
| stai:group | 46974.94 | 0.330 | 0.566 |
| group:block:lshs | 46973.65 | 0.712 | 0.399 |
| lshs:group | 46971.65 | 0.005 | 0.942 |
| block:lshs | 46970.61 | 0.956 | 0.328 |
| **group:block:sds** | 46972.46 | 3.849 | 0.050* |
| **lshs** | 46974.57 | 5.962 | 0.015 |
| **stai** | 46972.94 | 4.332 | 0.037 |

**Note.** Variables were removed in a stepwise fashion using likelihood ratio tests. Predictors were retained if their removal significantly worsened model fit (*p* < .05). Significant predictors retained in the final model are **bolded** and their *p*-values shown in red. * Value rounded up from 0.0498 and still meets statistical threshold.

**Table S2: Stepwise fixed effects selection process for the confidence model**

| Variable removed | AIC | χ² | *p*-value |
| --- | --- | --- | --- |
| None (full model) | 26887.13 | NA | NA |
| group:block:lshs | 26880.98 | 0.061 | 0.805 |
| lshs:group | 26876.23 | 0.021 | 0.886 |
| block:lshs | 26868.87 | 0.244 | 0.621 |
| group:block:stai | 26863.65 | 1.215 | 0.270 |
| block:stai | 26855.85 | 0.012 | 0.911 |
| stai:group | 26851.59 | 0.724 | 0.395 |
| **group:block:sds** | 26849.86 | 4.808 | 0.028 |
| **lshs** | 26854.94 | 9.646 | 0.002 |
| **stai** | 26863.07 | 17.916 | < .001 |

**Note.** Variables were removed in a stepwise fashion using likelihood ratio tests. Predictors were retained if their removal significantly worsened model fit (*p* < .05). Significant predictors retained in the final model are **bolded** and their *p*-values shown in red.

**5.2 Control analysis: Conditioned hallucination confidence effects persist after controlling for response rates**

Given that our confidence analysis focused on ‘yes’ trials, our model may be confounded by differing response rates: in short, the observed changes in confidence may be an artifact of changing response thresholds rather than changing confidence in conditioned hallucinations. To evaluate this influence, we computed overall and block-by-block response rates for each participant and included them as covariates in the final models. We found that both were significantly related to confidence ratings, with higher response rates predicting higher confidence in conditioned hallucinations (overall response rate: β = 1.092, SE = 0.382, χ²(1) = 8.05, p = .005; block response rate: β = 0.775, SE = 0.079, χ²(1) = 95.47, p < .001). However, all fixed effects of interest remained significant even after partialling these response rates out. Specifically, we found that in these updated models the three-way interaction of spider phobia group and block (overall: β = −0.093, SE = 0.042, χ²(1) = 4.96, p = .026; block: β = −0.083, SE = 0.041, χ²(1) = 4.13, p = .042), the main effect of hallucination-proneness (overall: β = 0.140, SE = 0.049, χ²(1) = 8.21, p = .004; block: β = 0.143, SE = 0.048, χ²(1) = 8.69, p = .003) and the main effect of trait anxiety (overall: β = −0.194, SE = 0.048, χ²(1) = 16.07, p < .001; block: β = −0.196, SE = 0.047, χ²(1) = 16.74, p < .001) remained significant predictors of confidence in conditioned hallucinations. These results indicate that any indirect influence of response rates on conditioned hallucination confidence is unlikely to account for our main findings.

**5.3 Model diagnostics**

Below we detail the model diagnostics for the final models to evaluate whether any of our findings could have been explained by violations in model assumptions. These were all carried out in the *R environment* [4]. The *performance* package [5] was used to investigate possible violations of all assumptions for the linear mixed effects models, while the *DHARMa* package [6] was used to simulate residuals and test model assumptions for the binomial generalized mixed-effects model in predicting response probability.

**5.3.1 Response model**

We first evaluate the assumptions of the response model for no-tone trials (conditioned hallucination reports), implemented as a binomial generalized mixed-effects model in the *lme4* [7] package. The model showed no underlying issues according to our diagnostic assessments (see Figure S3). Specifically, the Q-Q plot shows the simulated residuals are plotted in a straight line along the diagonal, and the KS test is non-significant. The model showed no problem with dispersion, given the bell-curved shape of the standard deviation of the simulated and fitted residuals, and non-significant dispersion test. There were no significant outliers according to the simulated residuals. The random effects for the intercepts and slopes for blocks showed normal distribution shapes. Overall, this suggests that the model is well fitted and our findings are unlikely to be contaminated by underlying violations in model assumptions.

**
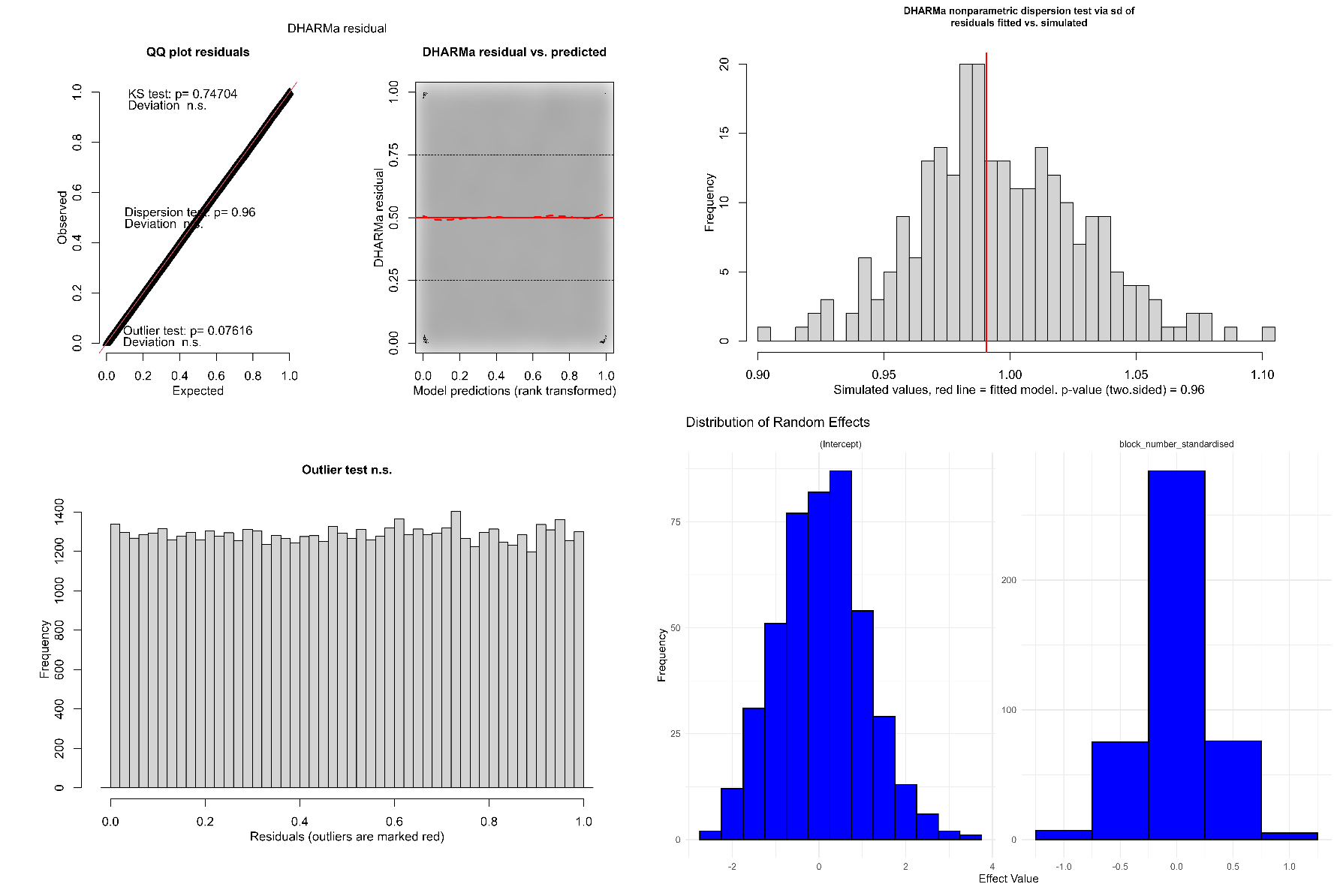
**

**Figure S3.** Diagnostic plots and test results for the binomial mixed effects model predicting response patterns on no-tone trials: top left, QQ plot of residuals; top middle, residuals vs. predicted values; top right, dispersion test; bottom left, outlier test; bottom right, distribution of random effects.

**5.3.2 Confidence model**

We next move on to our confidence model, which was modelled using a linear mixed effects model, once again using the lme4 package [7]. Unlike before, this model showed some issues in diagnostics (see Figure S4). A look at the residuals suggest that they show some deviations from normality, while there may also be some heterogeneity in variance across different levels of confidence. The posterior predictive check suggests that the observed data is not well predicted from the model. While the model assumes a single Gaussian distribution for the data, the observed data is instead in the shape of five smaller Gaussians. We reasoned that this lack of correspondence is likely driven by the fact that confidence responses are measured as integers from 1 to 5, while model predictions are estimated on a continuous scale, meaning that values between the integers will not be well captured by the model. Overall, this indicates a possible model misspecification, which could influence our findings.

One potential way to address this is to model confidence as an ordinal outcome. This models confidence as an ordered category of integers, rather than a single continuous outcome. Using this model, we aimed to see whether modelling the data in this way would lead to the same findings. This would tell us whether the possible model misspecification may have biased our findings. To do this, we conducted cumulative link mixed effects models as a robustness check. We conducted a frequentist version using the ordinal package [8] and a Bayesian version using the brms package [9,10] with Stan [11] using default weakly informative priors. We included both models since the frequentist version is more in line with our preregistered modelling approach in the paper but currently is unable to specify random slopes, while the Bayesian version allows one to specify both random intercepts and random slopes.

Based on these models, we found qualitatively the same findings as our original linear mixed effects model. For the frequentist cumulative link mixed effects model, all three fixed effects were significant: specifically, the three-way interaction of spider-phobia, group and block (β = −0.220, SE = 0.052, χ²(1) = 17.62, p < .001), and the main effects of trait anxiety (β = −0.406, SE = 0.111, χ²(1) = 13.11, p < .001) and hallucination-proneness (β = 0.287, SE = 0.112, χ²(1) = 6.46, p = .011) significantly improved model fit. Similarly, for the Bayesian model we found the three-way interaction of spider-phobia, group and block (β = -0.22, SE = 0.10, 95% CI [-0.42, -0.04]) and the main effects of hallucination-proneness (β = 0.31, SE = 0.12, 95% CI [0.08, 0.55]) and trait anxiety (β = -0.42, SE = 0.12, 95% CI [-0.66, -0.19]) all had 95% credible intervals which did not include 0. Model diagnostics for the Bayesian models indicated good convergence (all R̂ ≈ 1) and sufficient effective sample sizes (>1000). Posterior predictive checks showed that the ordinal model provided a better fit (see Fig. S5). Given that we find qualitatively the same findings as our linear model, we conclude that the potential model misspecification does not undermine the nature of our overall findings.


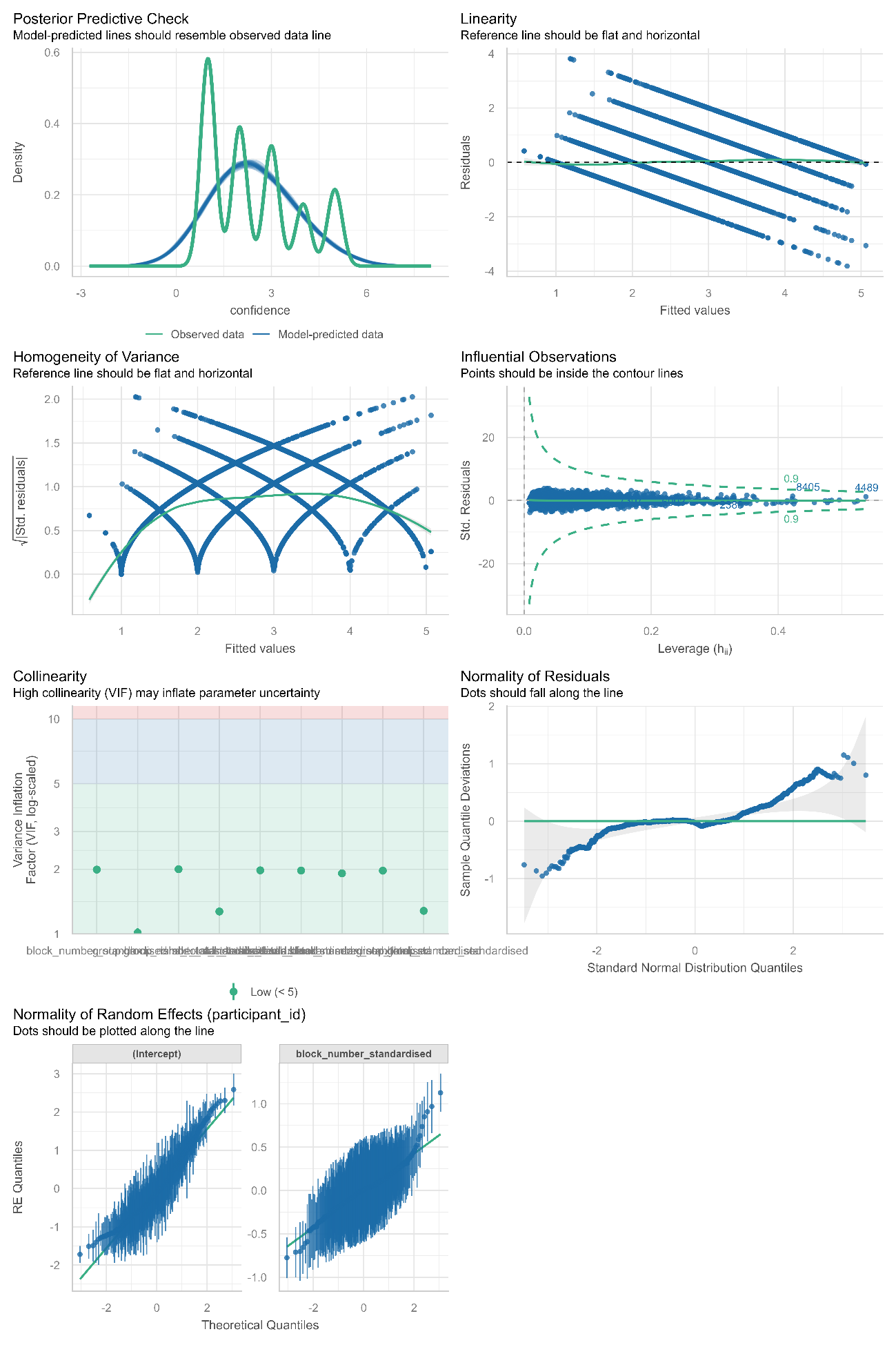


**Figure S4.** Model diagnostics for the linear mixed effects model predicting confidence. Panels show posterior predictive checks, residual diagnostics, collinearity (VIF), influential observations, and normality of random effects.


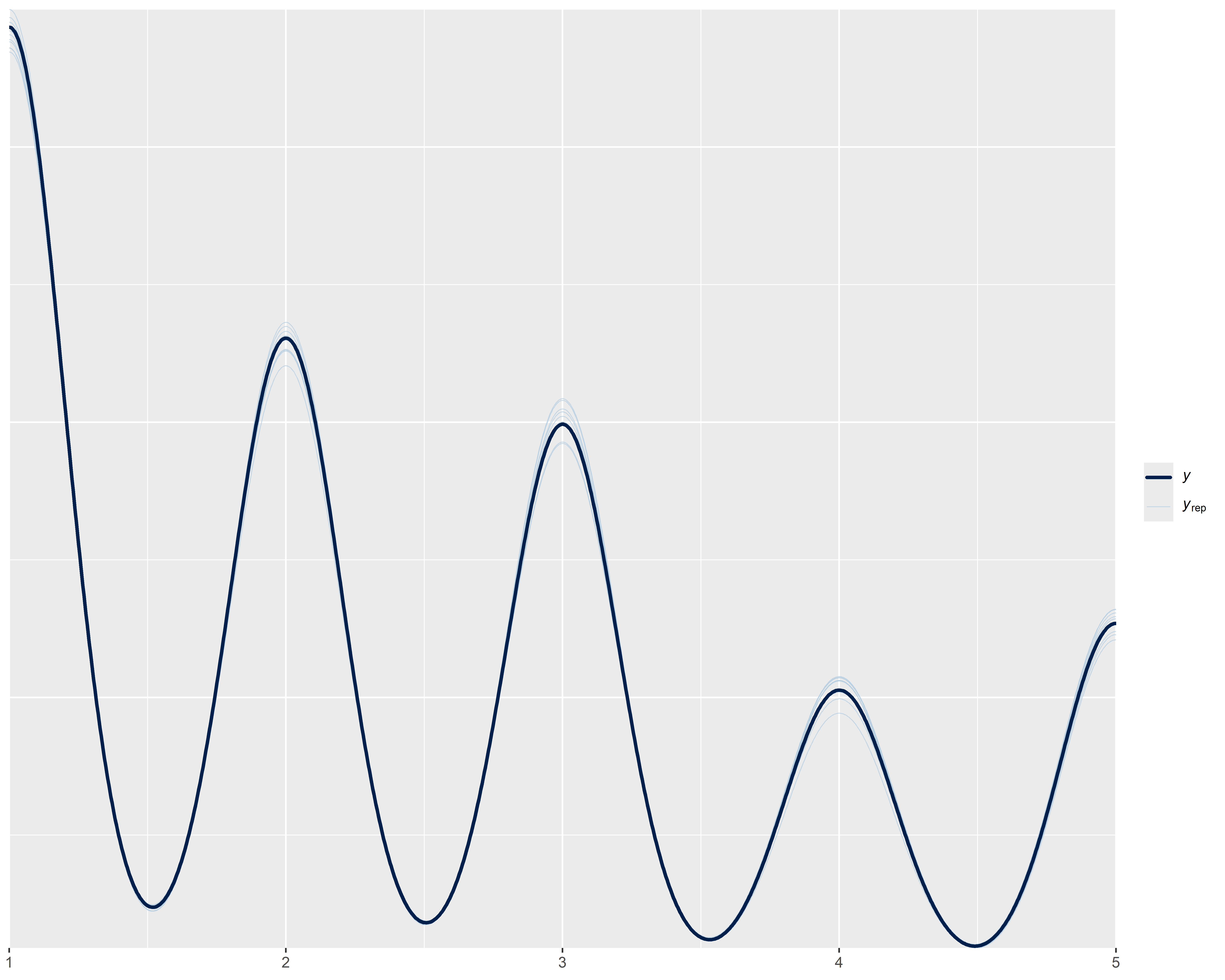


**Figure S5.** Posterior predictive check showing model-simulated data (y rep) overlaid on observed data (y) for the Bayesian cumulative link mixed effects model.

**6. Computational outcomes: Relating Predictors to the Weighting and Use of Prior Beliefs**

**6.1 Model diagnostics**

**6.1.1 Estimating hierarchical belief-weight trajectories**

We now move on to the models predicting outcome variables related to the hierarchical Gaussian filter model. We start with the modelling of belief-weights at the three hierarchical levels (X1, X2, X3, see Figures S6, S7 and S8). Visual inspection of the diagnostic plots indicates some issues with these models, such as non-normal residuals (X1, X2, X3), heterogeneity of variance (X1, X2, X3), influential observations (X2), non-linearity (X3) and high collinearity of predictors (X3).

We start with the perceptual belief-weight model (X1). For our three predictors, the original model found a significant effect for the three-way interaction (spider phobia × group × block) and hallucination-proneness, but an insignificant effect for trait anxiety. The model diagnostics suggest potential problems related to heteroskedasticity and non-normal residuals. Given that mixed effects models are generally robust to non-normal residuals [12], we focus our analysis on the influence of heteroskedasticity. To quantify the influence of heteroskedasticity, we reran the model using robust standard errors via the *clubSandwich* package [13]. This approach adjusts the standard errors to remain robust in the presence of non-constant residual variance. After applying this, we found that the three-way interaction was no longer significant (β = −0.009, SE = 0.005, *t*(231) = −1.818, *p* = 0.070), while the main effects of hallucination-proneness (β = 0.022, SE = 0.010, *t*(106) = 2.293, *p* = 0.021) and trait anxiety (β = −0.007, SE = 0.010, *t*(142) = −0.578, *p* = 0.491) were still consistent. We further investigated the two-way interaction of spider phobia and time separately in each group and found that it was significant for spiders (β = −0.010, SE = 0.004, *t*(110) = −2.470, *p* = .015), but not flowers (β = −0.001, SE = 0.003, *t*(128) = −0.381, *p* = 0.704). Thus, while there is robust evidence for a within-group effect for the spider group, the group difference implied by the three-way interaction is less robust, and may be influenced by the presence of heteroskedasticity.

We then moved on to the contingency belief-weight model (X2), where heteroskedasticity and non-normal residuals were once again potential issues; additionally, the model suggests there may be influential observations. To assess the potential bias of influential observations, we iteratively removed influential observations until all points fell within the contour lines (see Figure S9). Based on the updated model, we found the same effects as in the original model: the three-way interaction (β = −0.106, SE = 0.035; *t*(443) = -3.03, *p* = .003) and hallucination-proneness was significant (β = 0.133, SE = 0.054; *t*(441) = 2.45, *p* = .015), while trait anxiety remains non-significant (β = −0.069, SE = 0.054; *t*(441) = -1.26, *p* = .207). Therefore, our findings appear not to be affected by influential observations.

We then once again evaluated the potential influence of heteroskedasticity. The procedure was the same as the X1 model. Using the robust standard errors approach, we reproduced all of our findings from the original unadjusted model: the three-way interaction remained significant (β = −0.098, SE = 0.043, *t*(232) = −2.293, *p* = .023) and hallucination-proneness was also significant (β = 0.136, SE = 0.054, *t*(107) = 2.528, *p* = .013), while trait anxiety remained insignificant (β = −0.064, SE = 0.057, *t*(142) = −1.135, *p* = .258). Taken together with the fact that linear mixed-effects models are generally robust to moderate violations of normality [12], we interpret the findings from the X2 model to be robust to possible violations of model assumptions relating to heteroskedasticity and influential outliers.

We finally move on to the volatility belief-weighting model. The original model found no significant effect for any of the three predictors of interest. The model diagnostics suggest several major issues: there is evidence of non-linearity, heteroskedasticity, high collinearity, and a singular fit. We used a Box-Cox transformation using the MASS package [14] to find a possible suitable transformation for the data. This helped the non-linearity, the high collinearity, and the singular fit of the model; however, the model still showed some heteroskedasticity and non-normal residuals (see Figure S10). On the basis of the transformed model, we reran the analysis using robust standard errors. All predictors of interest remained non-significant: the three-way interaction (β = 0.001, SE = 0.004, *t*(232) = 0.341, *p* = .734), hallucination-proneness (β = −0.002, SE = 0.001, *t*(107) = −1.127, *p* = .262), and trait anxiety (β = 0.002, SE = 0.002, *t*(142) = 1.167, *p* = .245). Therefore, even after suitable robustness checks there are no significant effects of our fixed effects of interest in predicting volatility belief-weights.


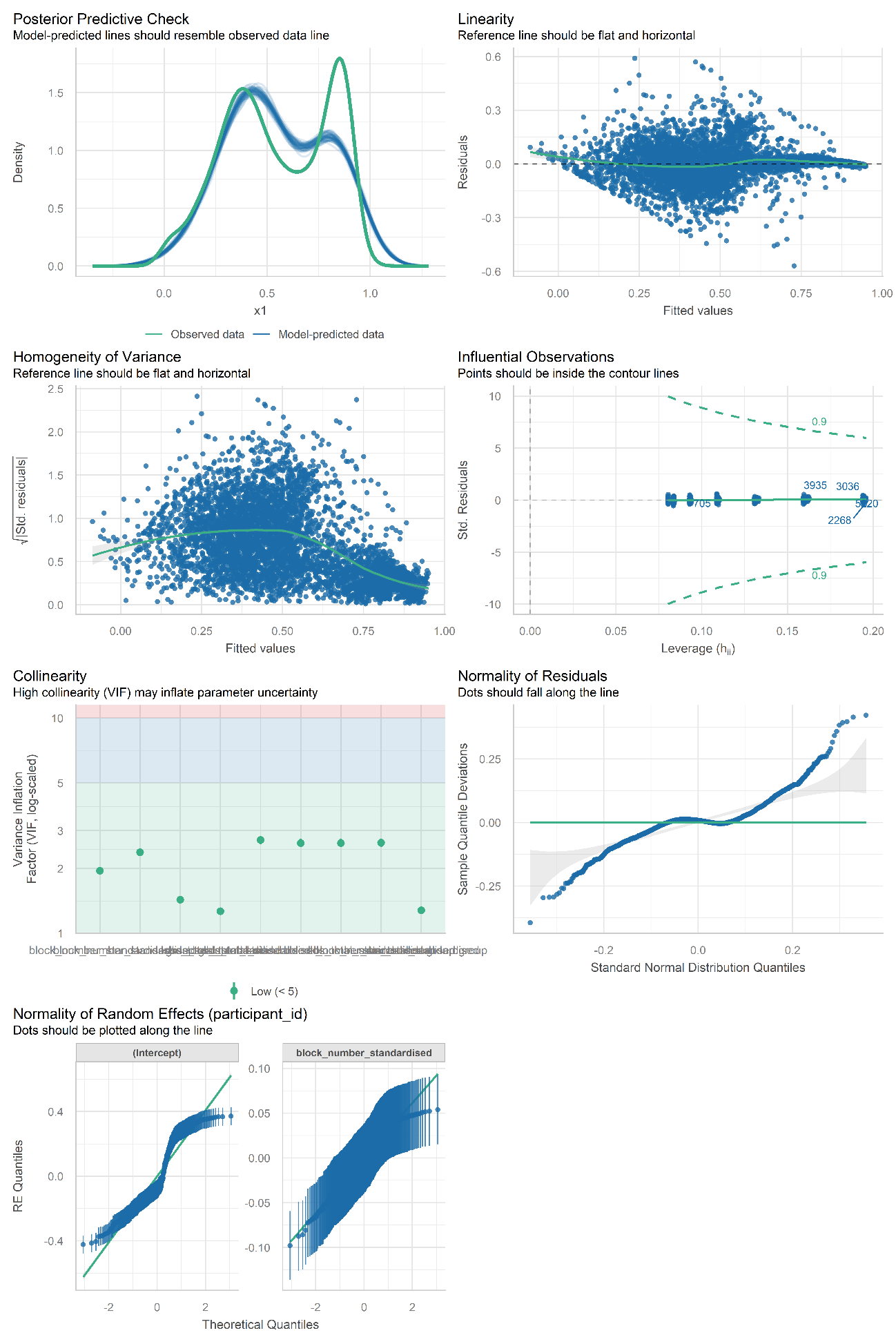


**Figure S6.** Model diagnostics for the linear mixed effects model predicting perceptual belief weighting (X1). Panels show posterior predictive checks, residual diagnostics, collinearity (VIF), influential observations, and normality of random effects.


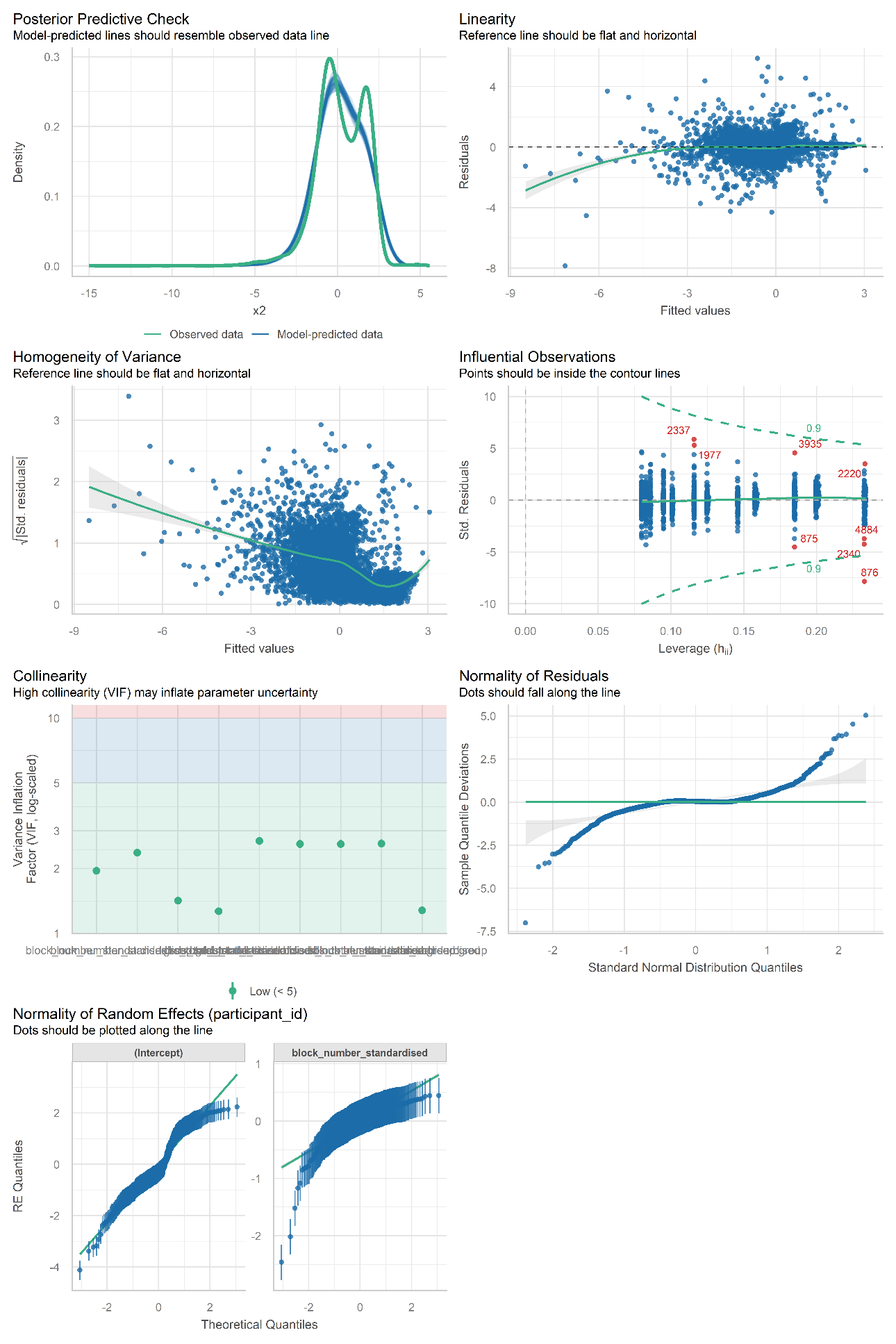


**Figure S7.** Model diagnostics for the linear mixed effects model predicting audiovisual contingency belief weighting (X2). Panels show posterior predictive checks, residual diagnostics, collinearity (VIF), influential observations, and normality of random effects.


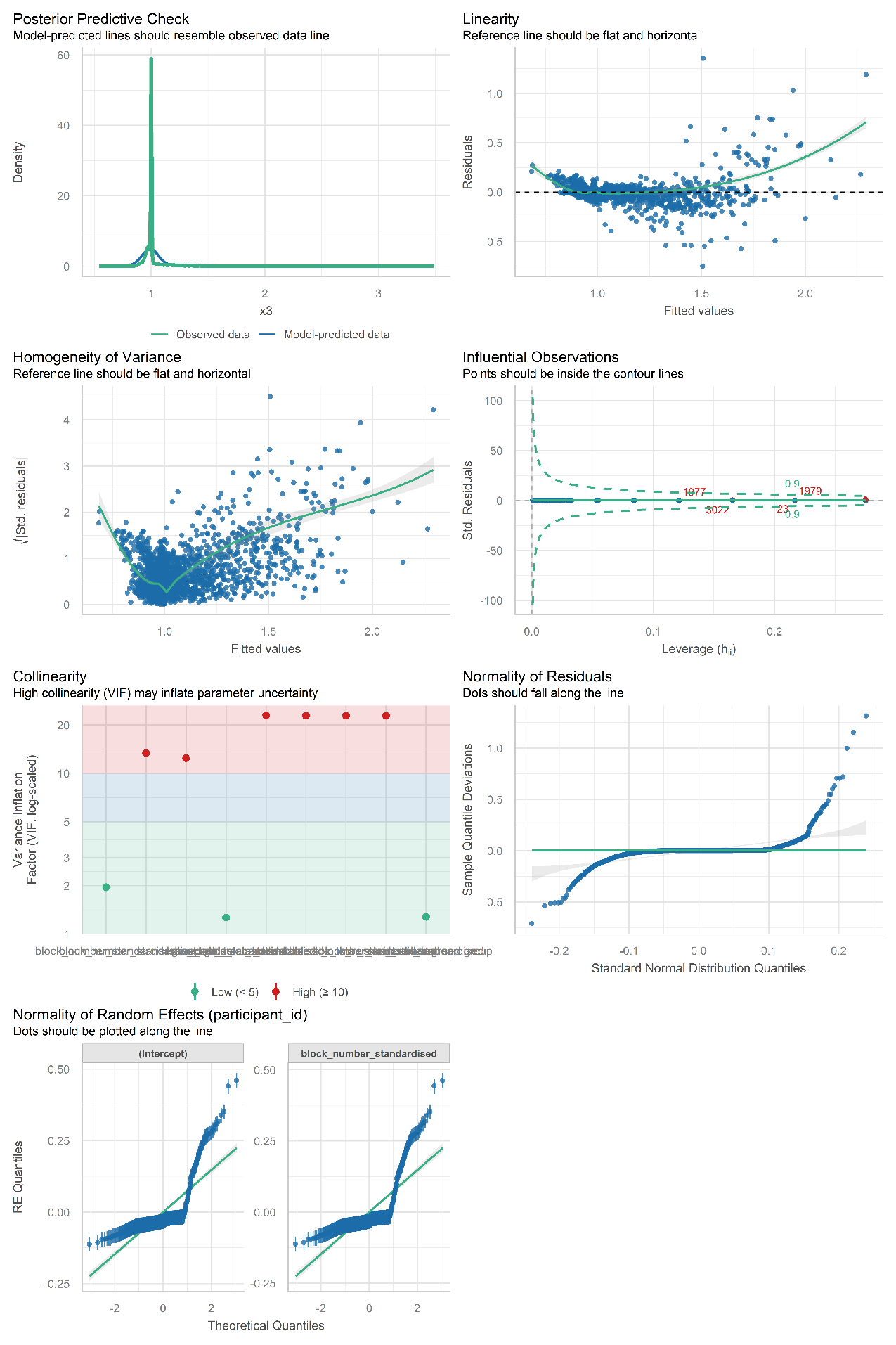


**Figure S8.** Model diagnostics for the linear mixed effects model predicting volatility of contingencies belief weighting (X3). Panels show posterior predictive checks, residual diagnostics, collinearity (VIF), influential observations, and normality of random effects.

**
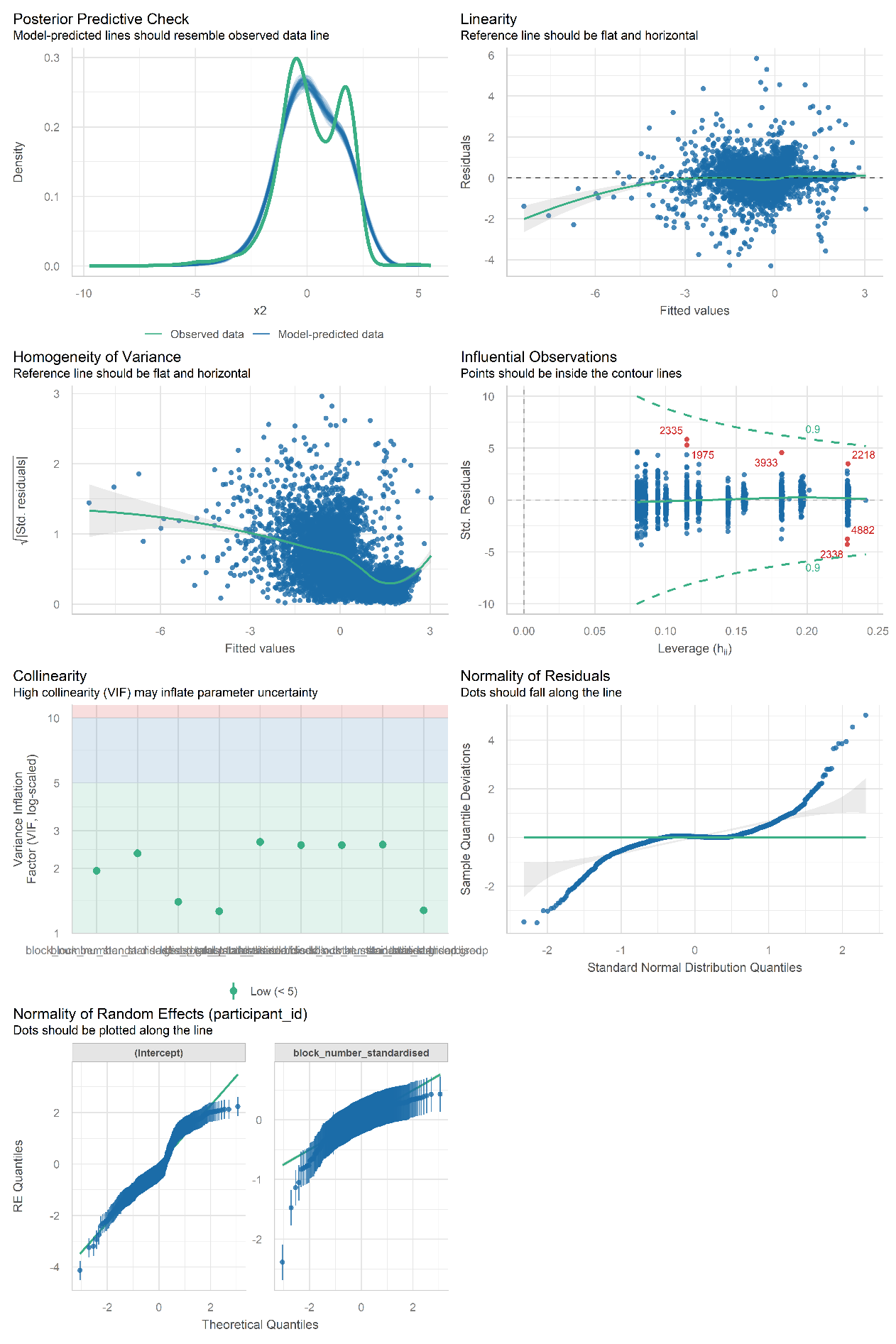
**

**Figure S9.** Model diagnostics for a linear mixed effects model predicting contingency belief weighting (X2) after outlier removal, based on the top 5% according to cook’s distance. Panels show posterior predictive checks, residual diagnostics, collinearity (VIF), influential observations, and normality of random effects.

**
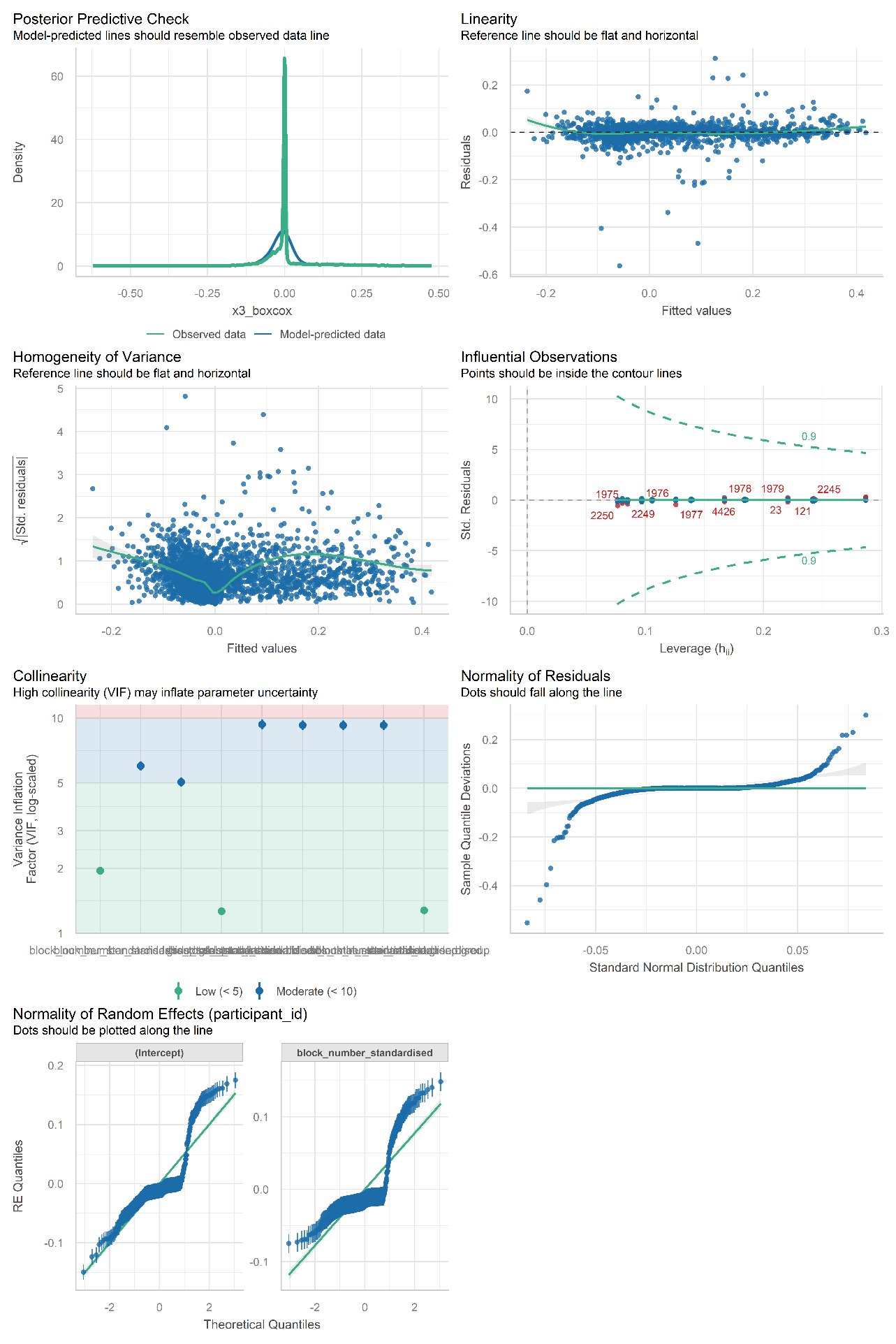
Figure S10.** Model diagnostics for the Box-Cox transformed linear mixed effects model predicting volatility belief weighting (X3). Panels show posterior predictive checks, residual diagnostics, collinearity (VIF), influential observations, and normality of random effects.

**6.1.2 Estimating participant-level parameters**

We next move on to our parameters fit at the participant level: the relative weighting of priors and sensory evidence (*v*), decision noise (ζ), and transition probabilities between the belief levels one and two (ω₂) and two and three (ω₃).

The untransformed prior-weighting (*ν)* model found a significant effect for the interaction of spider phobia and group (β = 0.167, SE = 0.070, χ²(1) = 5.69, *p* = .017) and trait anxiety (β = –0.084, SE = 0.039, χ²(1) = 4.55, *p* = .033), but not hallucination-proneness (β = 0.061, SE = 0.039, χ²(1) = 2.46, *p* = .117); the untransformed decision noise model found a significant effect for the interaction between spider phobia and group (β = 0.756, SE = 0.348, χ²(1) = 4.75, p = .029) but not hallucination proneness (β = 0.119, SE = 0.194, χ²(1) = 0.38, p = .538) nor trait anxiety (β = −0.140, SE = 0.195, χ²(1) = 0.52, p = .472); the omega models found no significant predictor at any level: for ω₂, spider phobia × group (β = 0.180, SE = 0.459, χ²(1) = 0.16, p = .692), hallucination-proneness (β = −0.183, SE = 0.256, χ²(1) = 0.52, p = .471), and trait anxiety (β = −0.133, SE = 0.257, χ²(1) = 0.27, p = .604) were all nonsignificant; for ω₃, spider phobia × group (β = 0.050, SE = 0.044, χ²(1) = 1.33, p = .248), hallucination-proneness (β = 0.023, SE = 0.024, χ²(1) = 0.87, p = .350), and trait anxiety (β = −0.011, SE = 0.025, χ²(1) = 0.22, p = .640) were likewise nonsignificant.

Based on the diagnostics of these models, the originally fitted models indicate poor model fit, with observable discrepancies between the model predictions and observed data, large deviations from normality for residuals, and an overall suggestion of a non-linear shape (see Figures S11- S14). To improve model fit and account for potential non-normality in the data, we applied Box–Cox transformations to all these outcome variables.

Following the transformations, we replotted the model diagnostics (see Figures S15-S18). The posterior predictive checks for both prior-weighting (*v)* and decision noise (ζ) models were much improved and reproduced the general shape of the observed data. Furthermore, deviations from normality for these two variables were reduced (albeit not perfectly). For the transition probability models, the transformations did not help address the underlying assumption issues (see Figures S17 and S18); in particular, omega2 appears to have a bimodal distribution and likely needs to be modelled using a different model family. However, given that both of these variables are not central to our hypotheses and showed no clear effects, we do not pursue these outcome variables any further.

Based on the updated Box–Cox transformation, we reran the prior weighting and decision noise models to see if we could reproduce our previous findings. For the prior weighting (*ν*) model, we found that the spider phobia × group interaction effect was no longer significant (β = 0.071, SE = 0.098, χ²(1) = 0.64, *p* = .423), while hallucination-proneness emerged as significant (β = 0.125, SE = 0.050, χ²(1) = 6.41, *p* = .011); trait anxiety remained significant from the original untransformed model (β = –0.118, SE = 0.050, χ²(1) = 5.65, *p* = .017). For the decision noise (ζ) model, all of the predictors were no longer significant: hallucination-proneness (β = 0.005, SE = 0.017, χ²(1) = 0.08, p = .774), trait anxiety (β = 0.002, SE = 0.018, χ²(1) = 0.02, p = .895), and the spider phobia × group interaction (β = 0.048, SE = 0.031, χ²(1) = 2.38, p = .123).

Given the uncertainty regarding the significance of these results, as a final robustness check we fit Bayesian models with weakly informative priors to compare 95% credible intervals for coefficients of interest and to assess model fit across transformed and untransformed outcomes. For prior weighting (*ν*), the Box–Cox transformed model reproduced significant effects of hallucination-proneness (β = 0.12, SE = 0.05, 95% CI [0.03, 0.22]) and trait anxiety (β = –0.12, SE = 0.05, 95% CI [–0.22, –0.02]), while the interaction of spider phobia and group was not significant (β = 0.07, SE = 0.09, 95% CI [–0.10, 0.24]). For decision noise (ζ), the Box-Cox transformed model reproduced the non-significant results of the frequentist model, with 95% credible intervals including zero (hallucination-proneness: β = 0.00, SE = 0.02, 95% CI [–0.03, 0.04]; trait anxiety: β = 0.00, SE = 0.02, 95% CI [–0.03, 0.04]; interaction of spider phobia and group: β = 0.05, SE = 0.03, 95% CI [–0.01, 0.11]). Plots for posterior predictive checks suggest that the Box-Cox transformed model is a better fit of the data than the untransformed one (see Figure S19). Diagnostics across models showed good convergence (R̂ ≈ 1.00) and sufficient effective sample sizes (> 1000) for our key parameters of interest. Given the agreement of the frequentist and Bayesian models, we conclude that hallucination-proneness and trait anxiety are significantly associated with an increased and decreased reliance on prior beliefs during the task respectively, while there are no robust relationships found between the fixed effects and changes in decision noise.

**
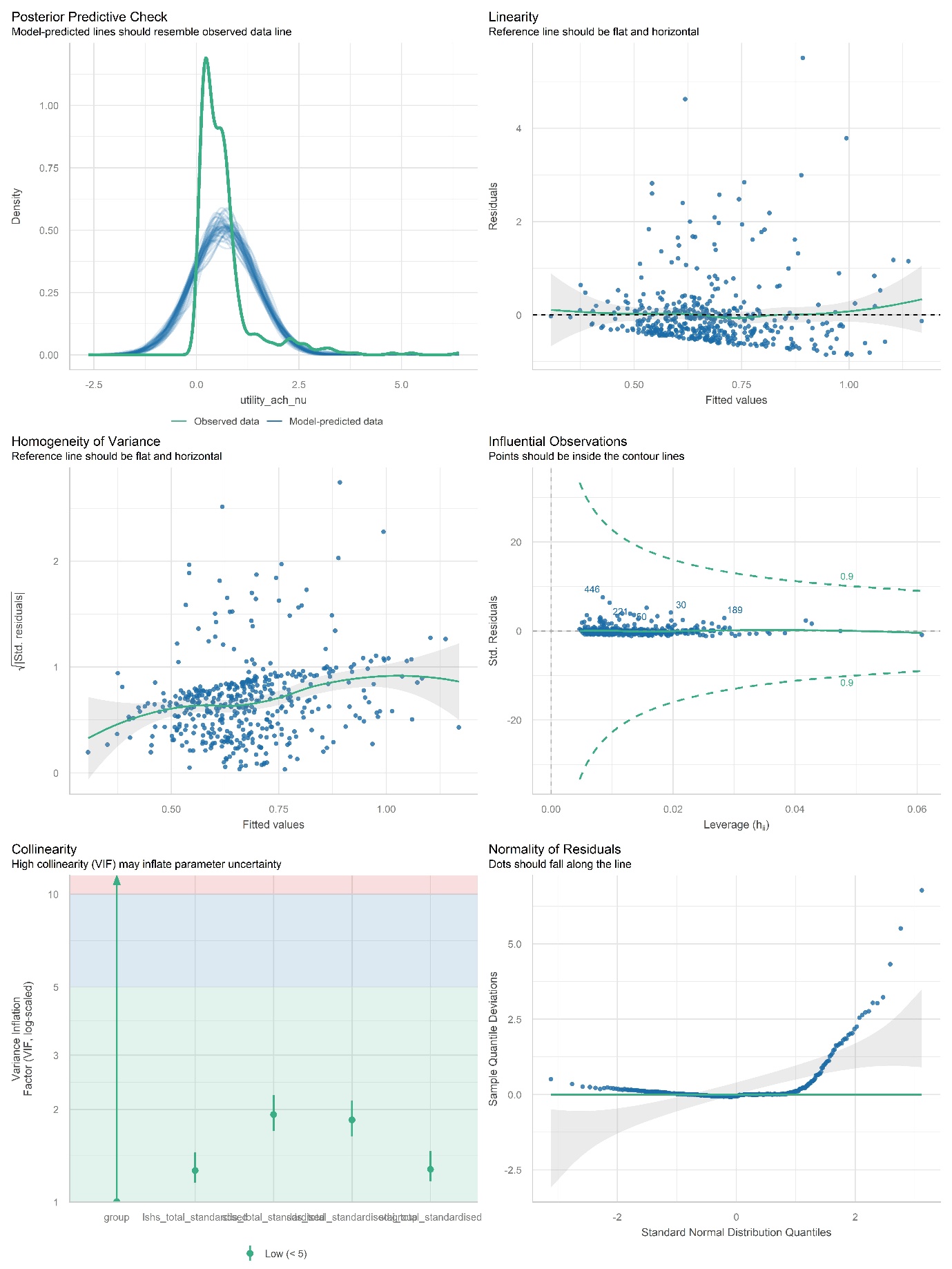
**

**Figure S11.** Model diagnostics for the untransformed linear model predicting relative weighting of prior beliefs and sensory evidence (*v*). Panels show posterior predictive checks, residual diagnostics, collinearity (VIF), influential observations, and normality of random effects.

**
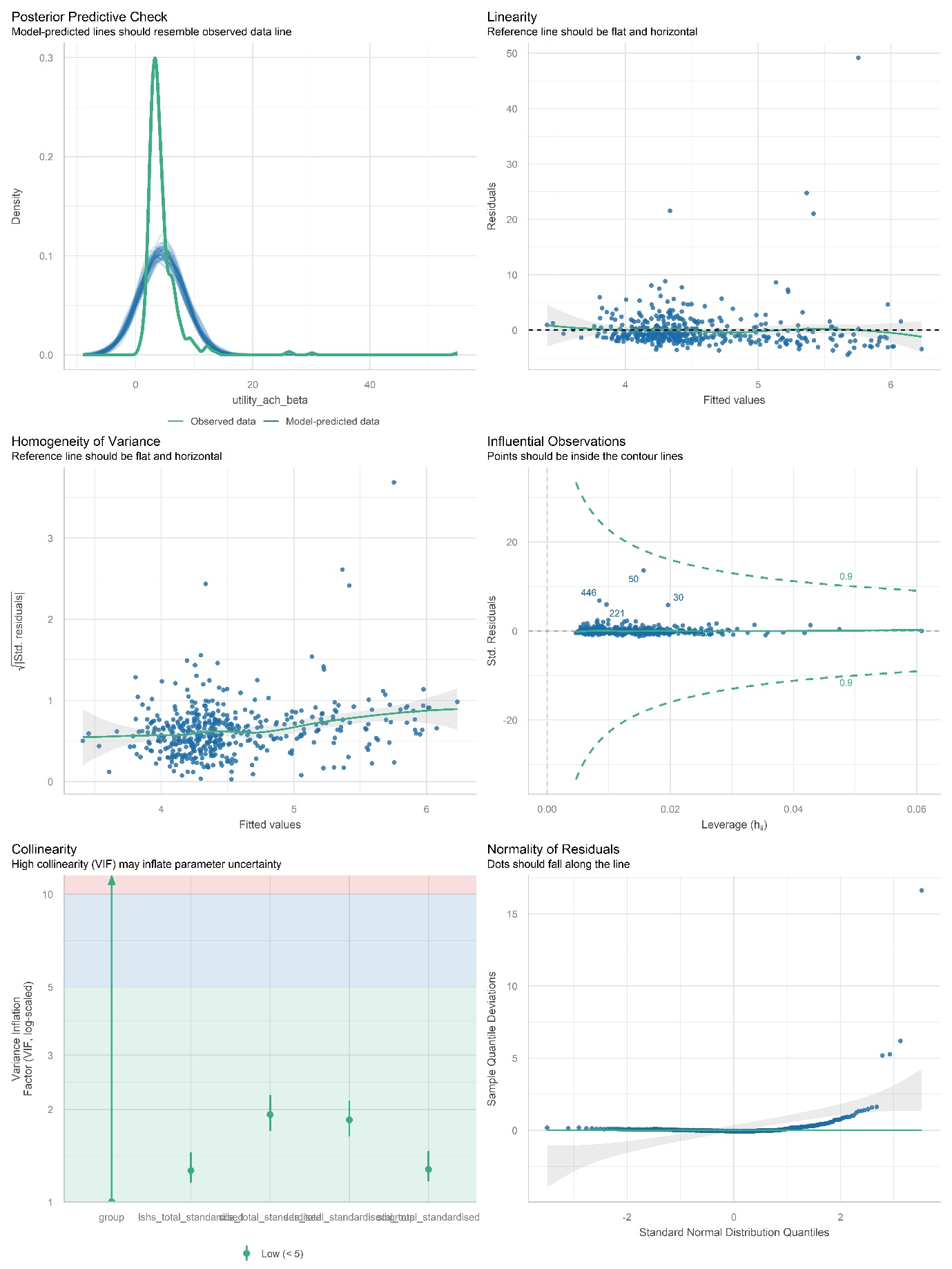
Figure S12.** Model diagnostics for the untransformed linear model predicting decision noise (ζ). Panels show posterior predictive checks, residual diagnostics, collinearity (VIF), influential observations, and normality of random effects.

**
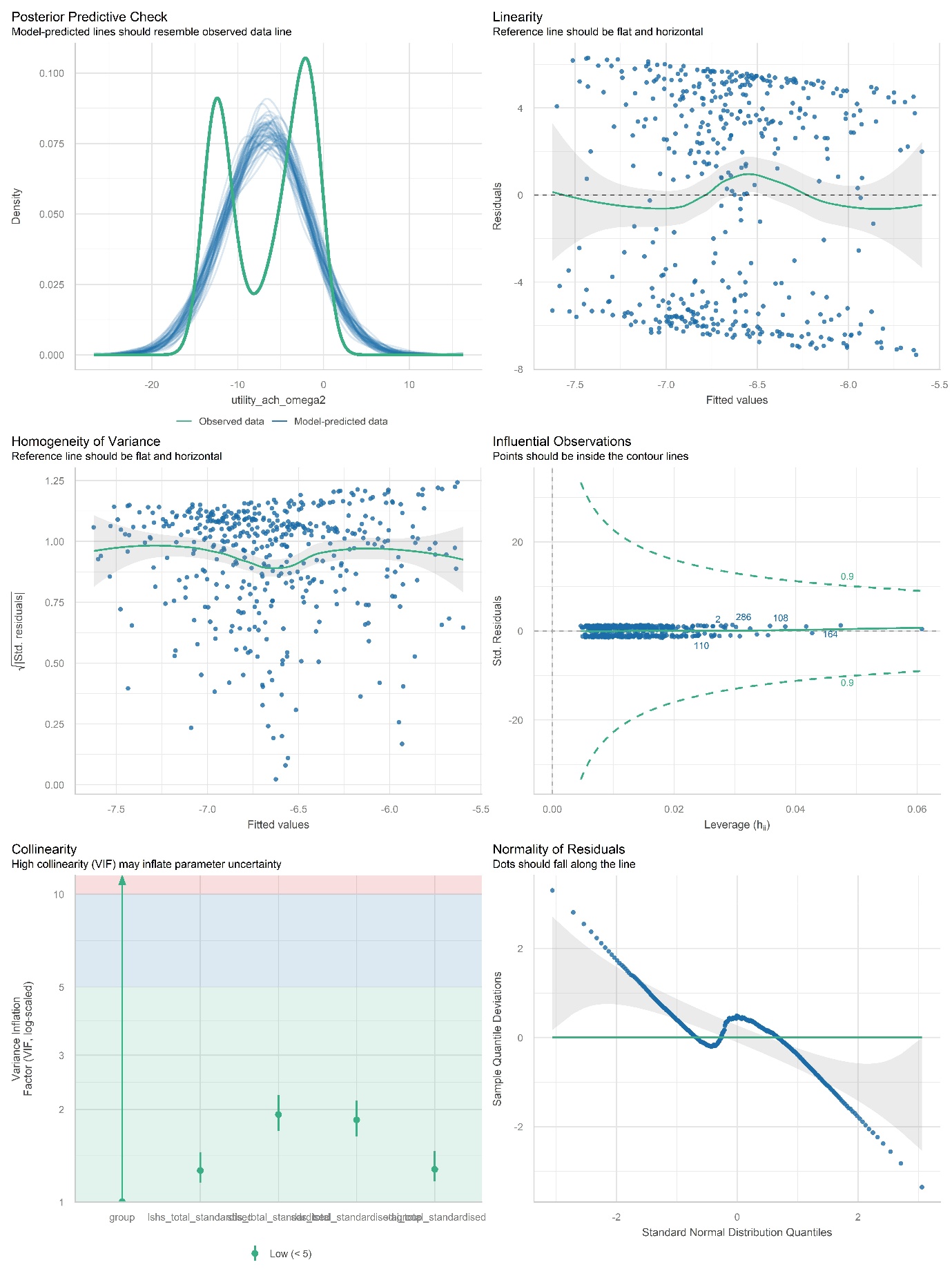
**

**Figure S13.** Model diagnostics for the untransformed linear model predicting transition probability between levels 1 and 2 (ω₂). Panels show posterior predictive checks, residual diagnostics, collinearity (VIF), influential observations, and normality of random effects.

**
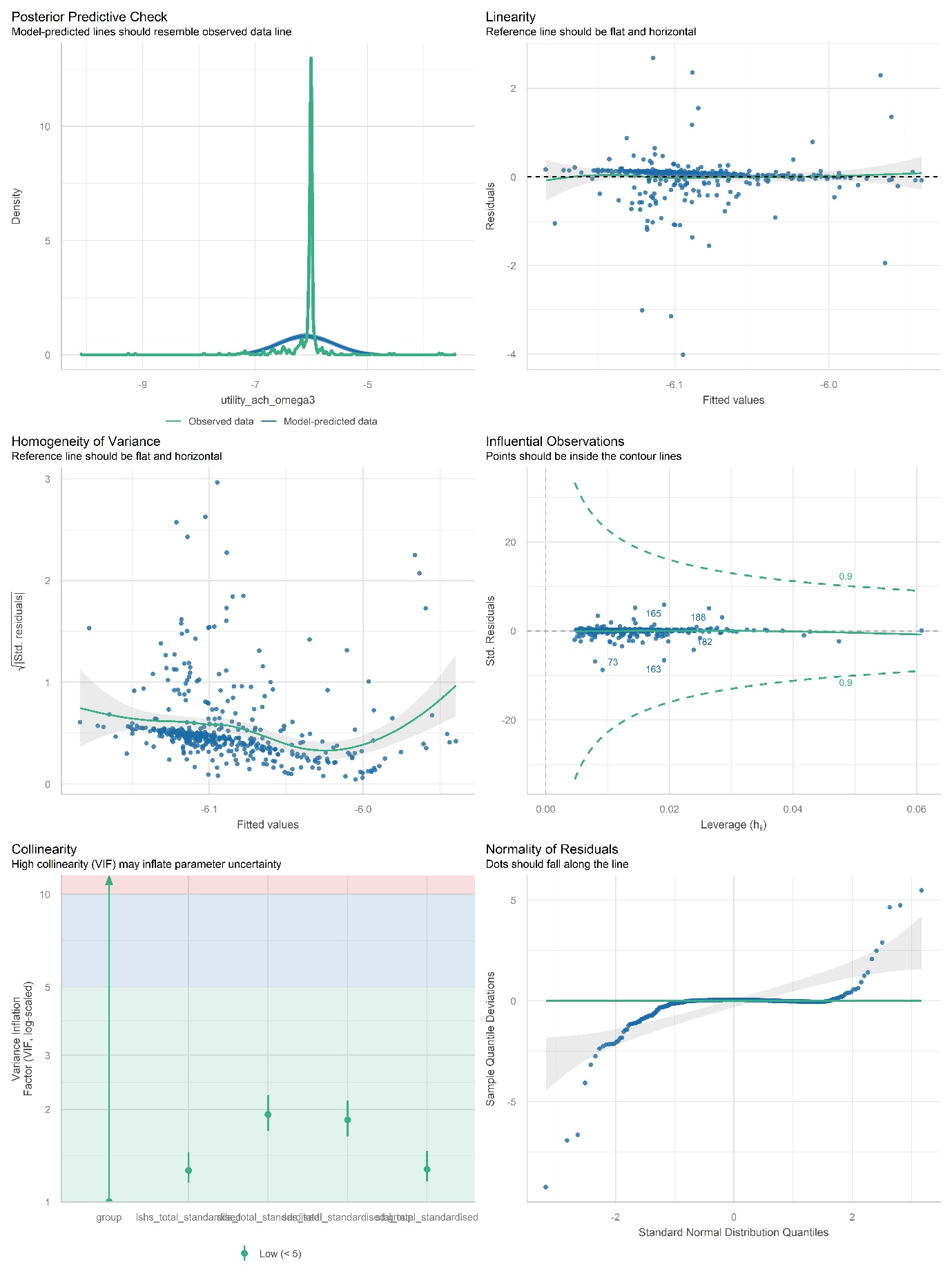
**

**Figure S14.** Model diagnostics for the untransformed linear model predicting transition probability between levels 2 and 3 (ω₃). Panels show posterior predictive checks, residual diagnostics, collinearity (VIF), influential observations, and normality of random effects.

**
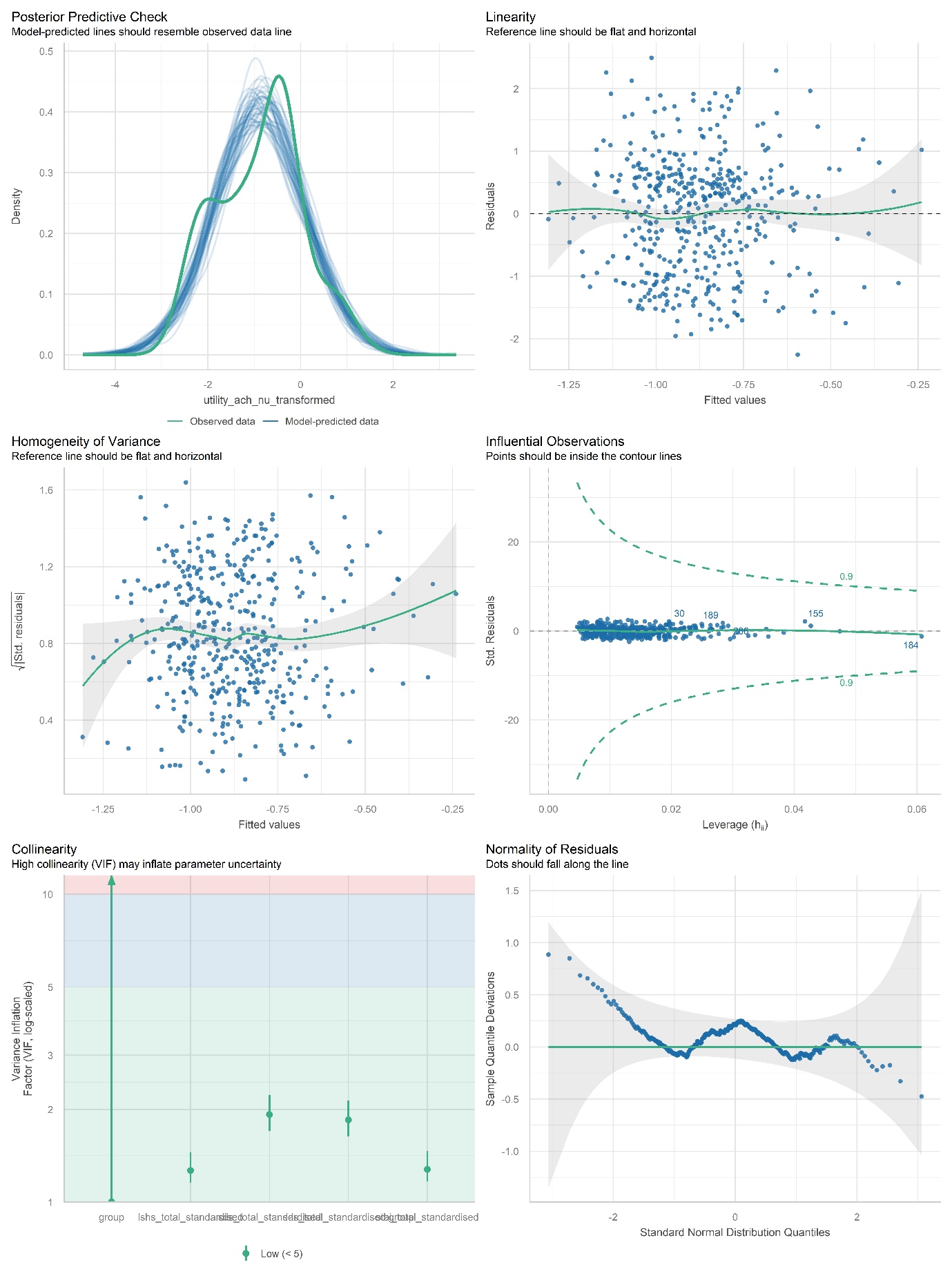
 Figure S15.** Model diagnostics for the Box-Cox transformed linear mixed effects model predicting relative weighting of prior beliefs and sensory evidence (*v*). Panels show posterior predictive checks, residual diagnostics, collinearity (VIF), influential observations, and normality of random effects.

**
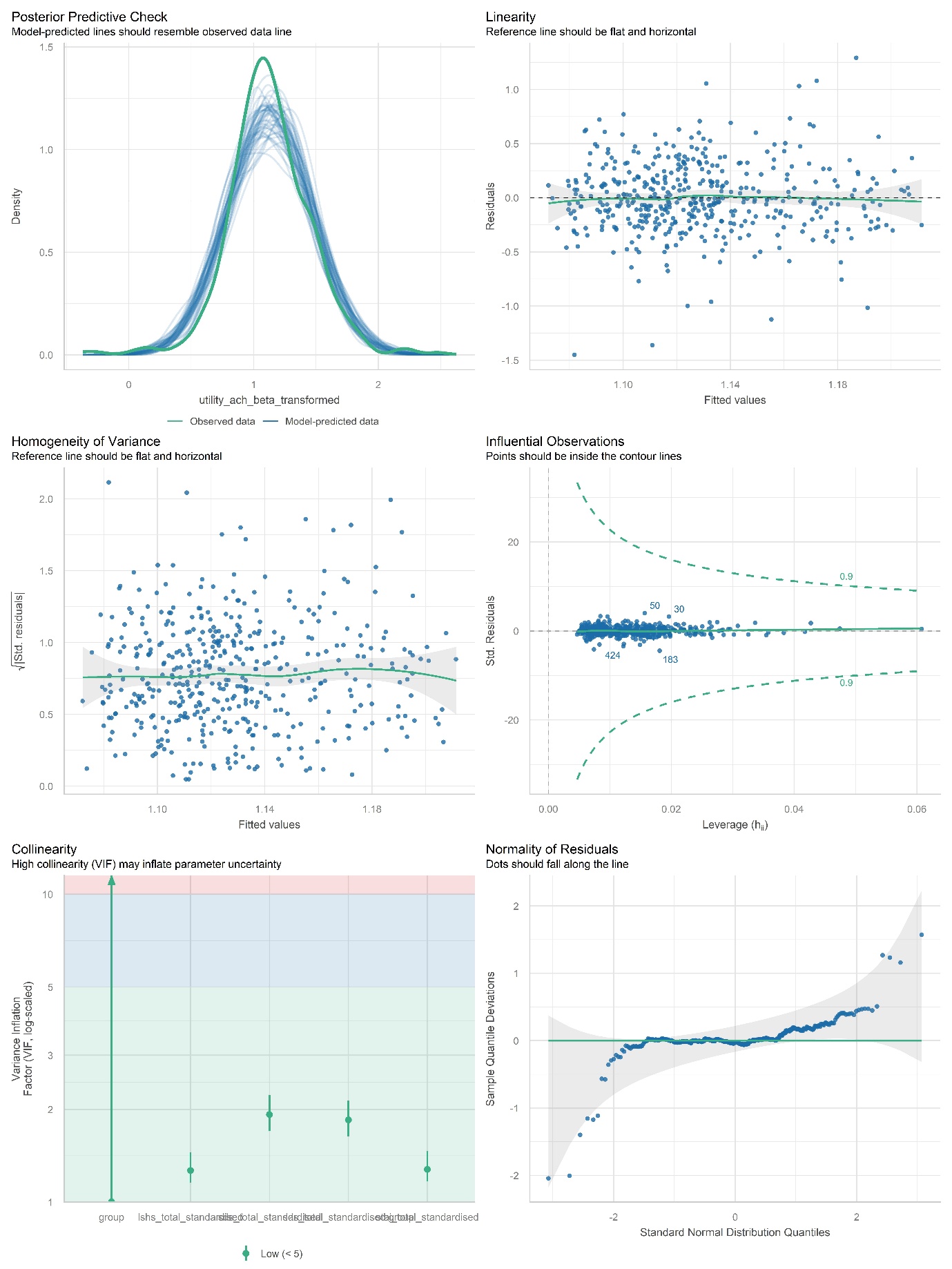
 Figure S16.** Model diagnostics for the Box-Cox transformed linear mixed effects model predicting decision noise (ζ). Panels show posterior predictive checks, residual diagnostics, collinearity (VIF), influential observations, and normality of random effects.

**
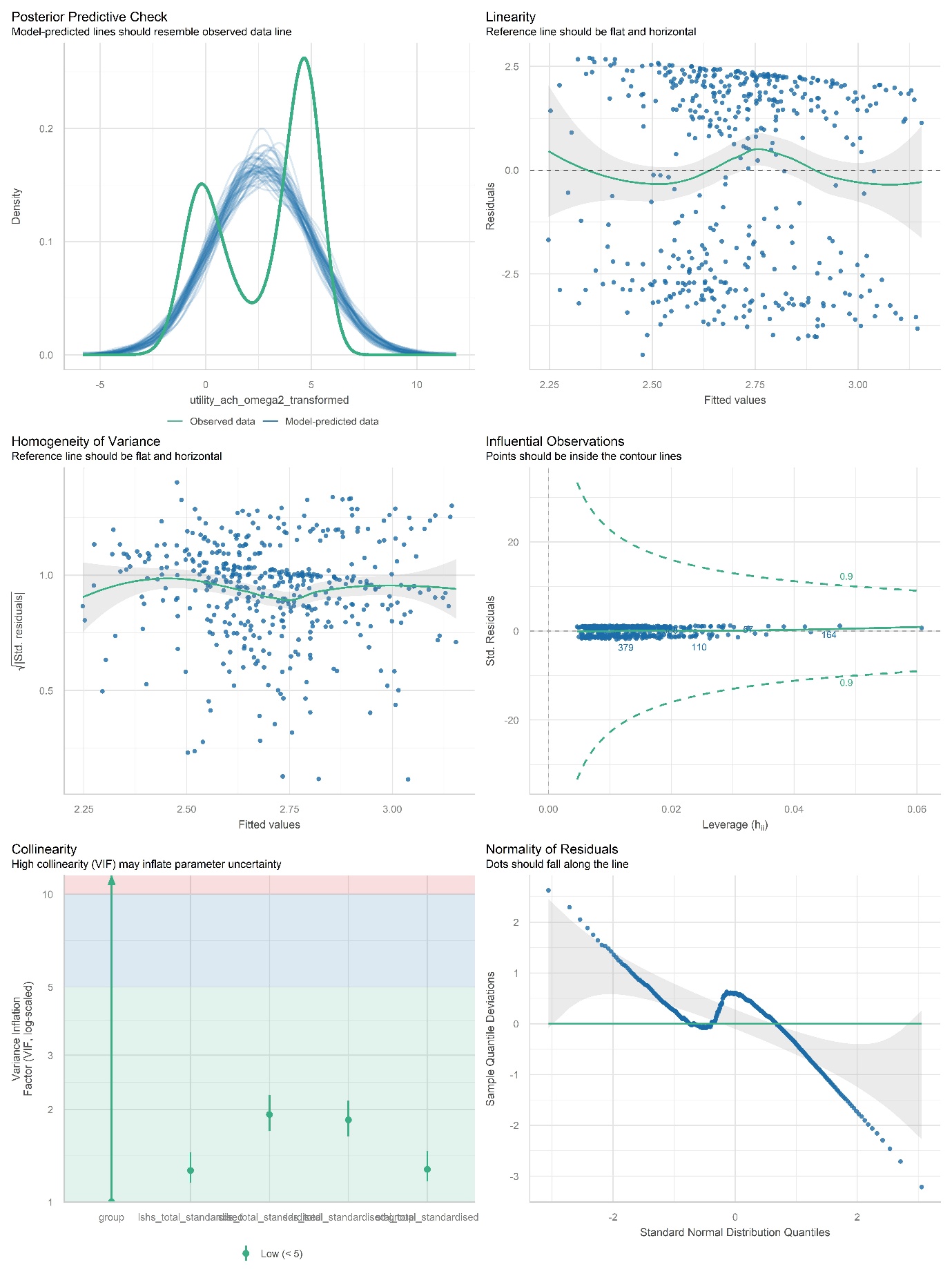
**

**Figure S17.** Model diagnostics for the Box-Cox transformed linear mixed effects model predicting transition probability between levels 1 and 2 (ω₂). Panels show posterior predictive checks, residual diagnostics, collinearity (VIF), influential observations, and normality of random effects.

**
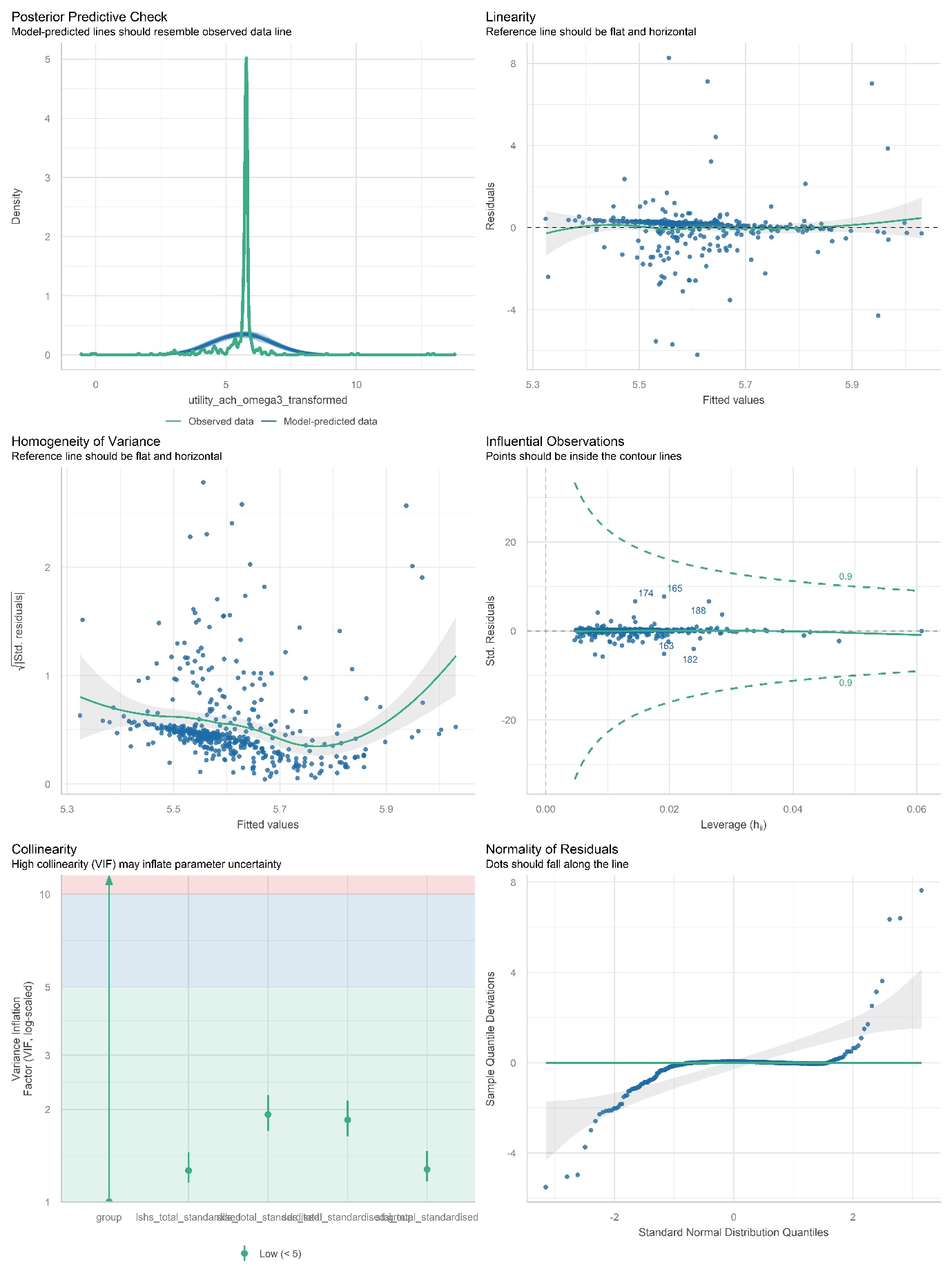
**

**Figure S18.** Model diagnostics for the Box-Cox transformed linear mixed effects model predicting transition probability between levels 2 and 3 (ω₃). Panels show posterior predictive checks, residual diagnostics, collinearity (VIF), influential observations, and normality of random effects.

**
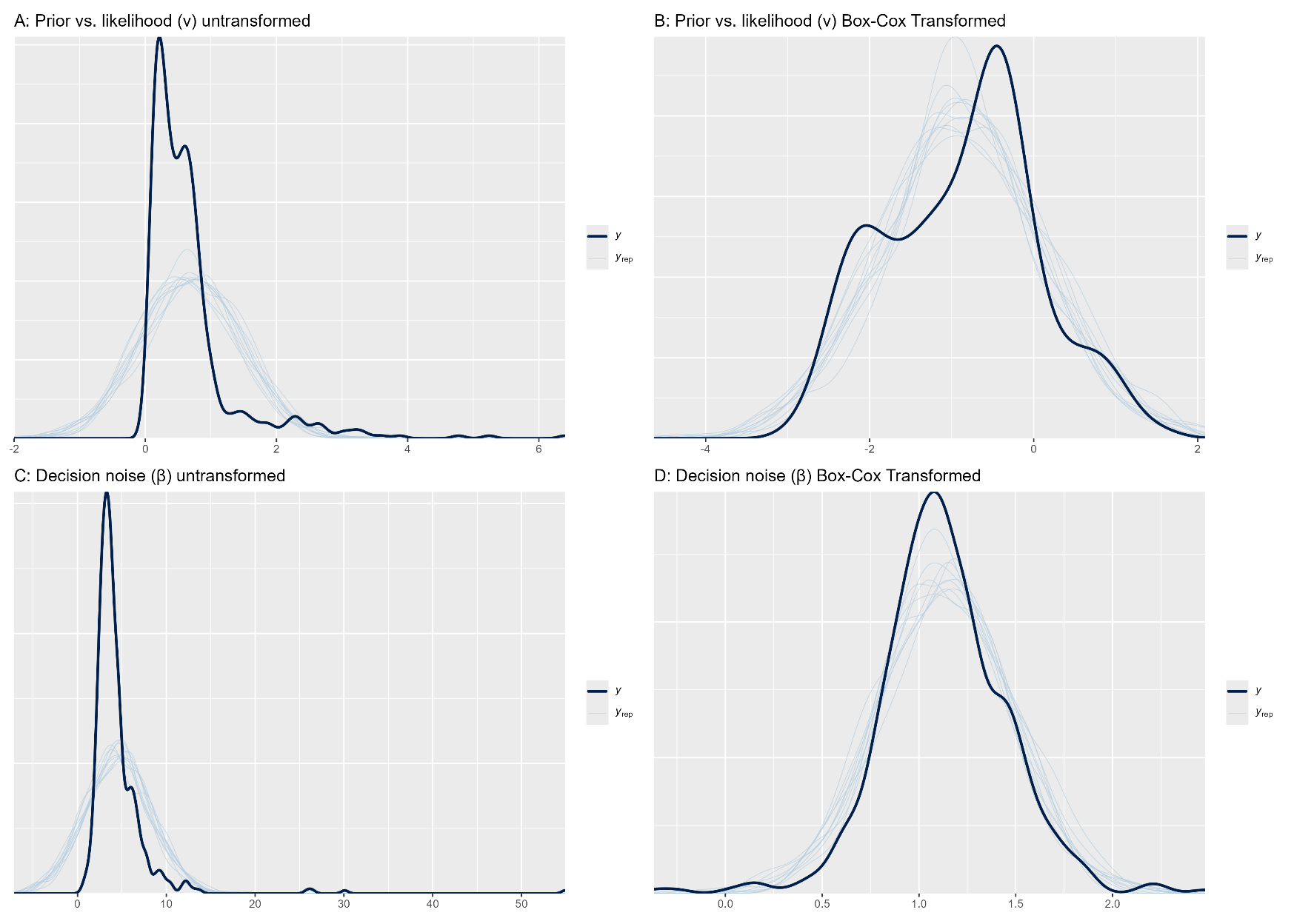
**

**Figure S19.** Posterior predictive checks comparing observed and predicted distributions for prior weighting (*v*) and decision noise (ζ), before (panels A and C) and after (panels B and D) Box-Cox transformation.

**7. Exploratory Analysis: Evaluating Non-linear Trajectories in Behavior and Belief-Weights**

**7.1 Evaluating non-linear changes in the flower images group**

This exploratory analysis evaluated non-linear changes in behavior and belief-weights. In the primary analysis, we focused on the spider images group to further investigate the time-dependent changes that were observed in our preregistered analyses. For completeness, we here present the corresponding results for the flower images group using the same analytic approach.

When estimating non-linear changes in the flower group, we found evidence for some non-linear interaction effects (see Figure S20); however, not to the extent of the spider group. Confidence models showed an insignificant non-linear effect based on all trials (edf = 1.80, F = 1.27, p = 0.182) and an insignificant linear effect on no-tone trials only (edf = 1.00, F = 0.83, p = 0.361). Response models found an insignificant non-linear effect across all trials (edf = 2.65, F = 1.70, p = .095), and a significant non-linear effect on no-tone trials (edf = 2.74, F = 7.19, p < .001). For the computational outcomes, significant non-linear interactions with spider phobia were observed for perceptual belief weights (X1: edf = 2.54, F = 4.39, p = .005) and for contingency belief weights (X2: edf = 2.45, F = 2.73, p = .033).

Overall, unlike the spider group, the flower group showed less consistent evidence for non-linear changes in behavior and belief-weights. Even where significant effects were observed, such as response tendencies on no-tone trials, the trajectories differed qualitatively from those in the spider group: the estimated effect of spider phobia peaked around block 8 in the flower group, whereas it was near its lowest point in the spider group. This pattern suggests that, although non-linear interactions may be present in both groups, their form and direction differ, indicating that they likely reflect group-specific rather than shared processes.

**
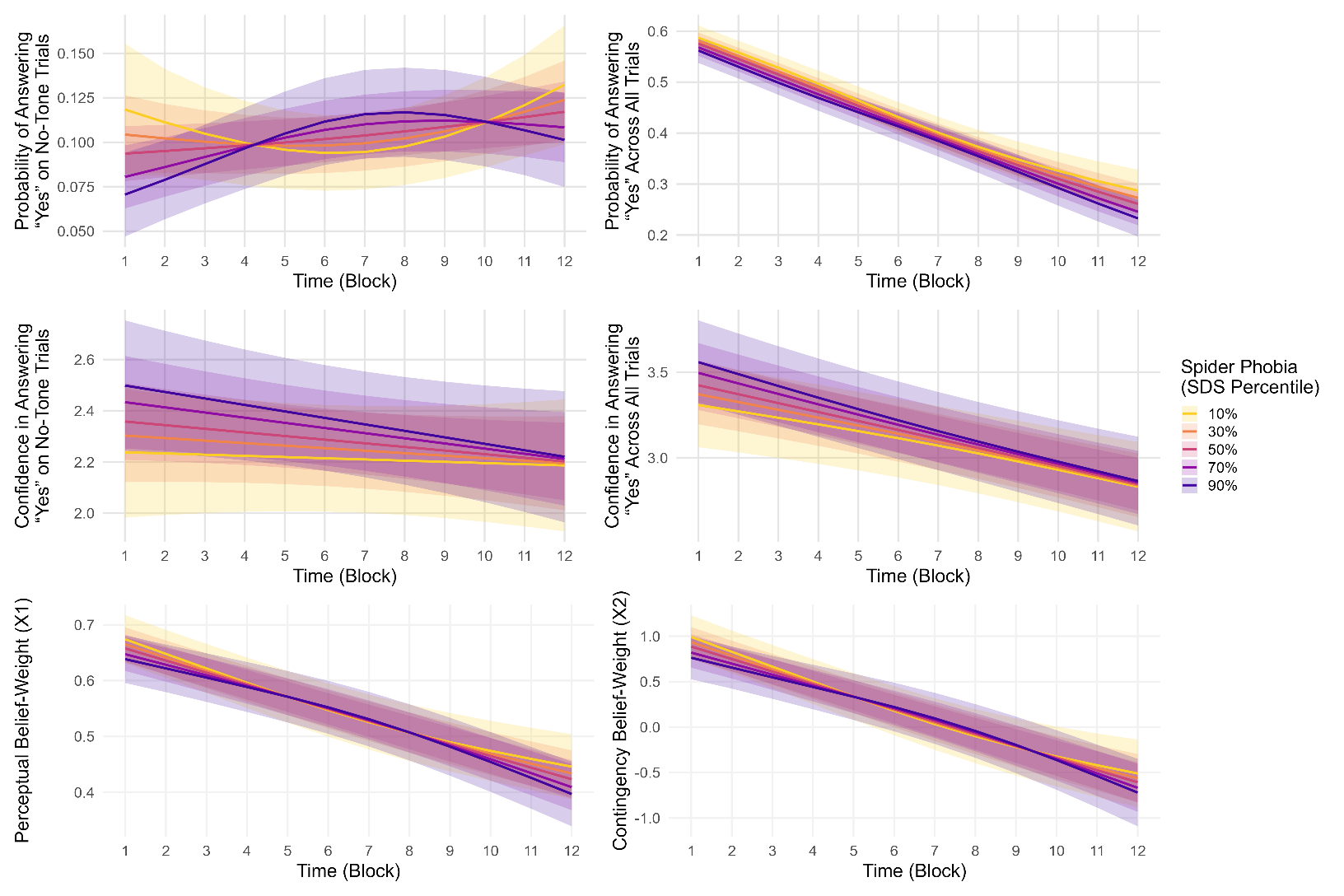
**

**Figure S20.** Model-predicted trajectories from generalized additive models estimating a smooth term for time (experimental block) in its interaction with spider phobia (SDS score) across six key outcomes in the flowers group. The top row shows the probability of reporting hearing a tone on no-tone trials (left) and across all trials (right). The middle row shows confidence in reporting tones on no-tone trials (left) and across all trials (right). The bottom row depicts perceptual (X1; left) and contingency belief-weights (X2; right). Lines represent predictions for five levels of spider phobia (10th to 90th percentile); shaded ribbons indicate 95% confidence intervals.

**7.2 Model diagnostics**

We finally evaluate diagnostics related to the generalized additive mixed models in the spider image group, implemented in the mgcv package [15]. The first set of these models were specified as an extension of our previous mixed-effects models by estimating a smooth term for experimental block in its interaction with spider phobia. Since these models were structurally equivalent to the previous mixed-effects models, with the exception of the estimation of a non-linear term, we focused our diagnostics on the estimation of this non-linear term. To do this we used the k-test from the mgcv package [15]. In generalized additive models, a k-test evaluates whether the effective degrees of freedom approach the upper limit of the specified basis dimension, with significant results indicating that the smooth may be underfitted due to an insufficient basis dimension. Note that k-index *p*-values are simulation-based and may differ slightly across runs; however, model estimates and fitted smooths are unaffected.

For all but one model, the k-index values were close to 1 with non-significant p-values, suggesting that the chosen basis dimension was adequate (response–all trials: k′ = 4, edf = 3.49, k-index = 0.99, p = .278; response–no-tone: k′ = 4, edf = 3.33, k-index = 0.98, p = .235; confidence–all trials: k′ = 4, edf = 3.44, k-index ≈ 1.00, p = .428; X1: k′ = 4, edf = 3.11, k-index = 0.97, p = .050; X2: k′ = 4, edf = 3.10, k-index = 0.97, p = .053). The only model that didn’t follow this pattern was the confidence no-tone model, which showed a low k-index and a significant p-value (k′ = 4, edf ≈ 1.00, k-index = 0.55, p < .001); however, as the estimated degrees of freedom was 1, indicating a linear relationship, this does not indicate a limitation of the chosen basis dimension. As a result, our results are unlikely to be determined by a basis dimension that is too restrictive.

**8. Exploratory Analyses: Relating Response Patterns to Changes in Sensitivity and Bias**

**8.1 Non-linear changes in sensitivity and bias in the flower images group**

The second set of generalized additive models related to evaluating non-linear changes in sensitivity (d′) and criterion (c) over time in relation to spider-phobia in the spider image group. For completeness, here in the supplementary material we present the corresponding results for the flower images group using the same analytic approach.

When estimating linear and non-linear changes in the flower group, we found no significant changes in signal detection parameters according to spider phobia over time. Specifically, for both sensitivity (d′) (25%: edf = 1.00, F = 0.73, p = .393; 50%: edf = 2.22, F = 3.27, p = .055; 75%: edf = 2.22, F = 2.03, p = .104), or decision criterion (c) (25%: edf = 2.37, F = 2.30, p = .060; 50%: edf = 2.34, F = 0.84, p = .312; 75%: edf = 1.62, F = 0.37, p = .564), no significant effects were found at any QUEST intensity levels. Furthermore, no consistent patterns were observed across the different levels (see Figure S21). This suggests that the observed pattern of non-linear changes in response bias is specific to the spider group.


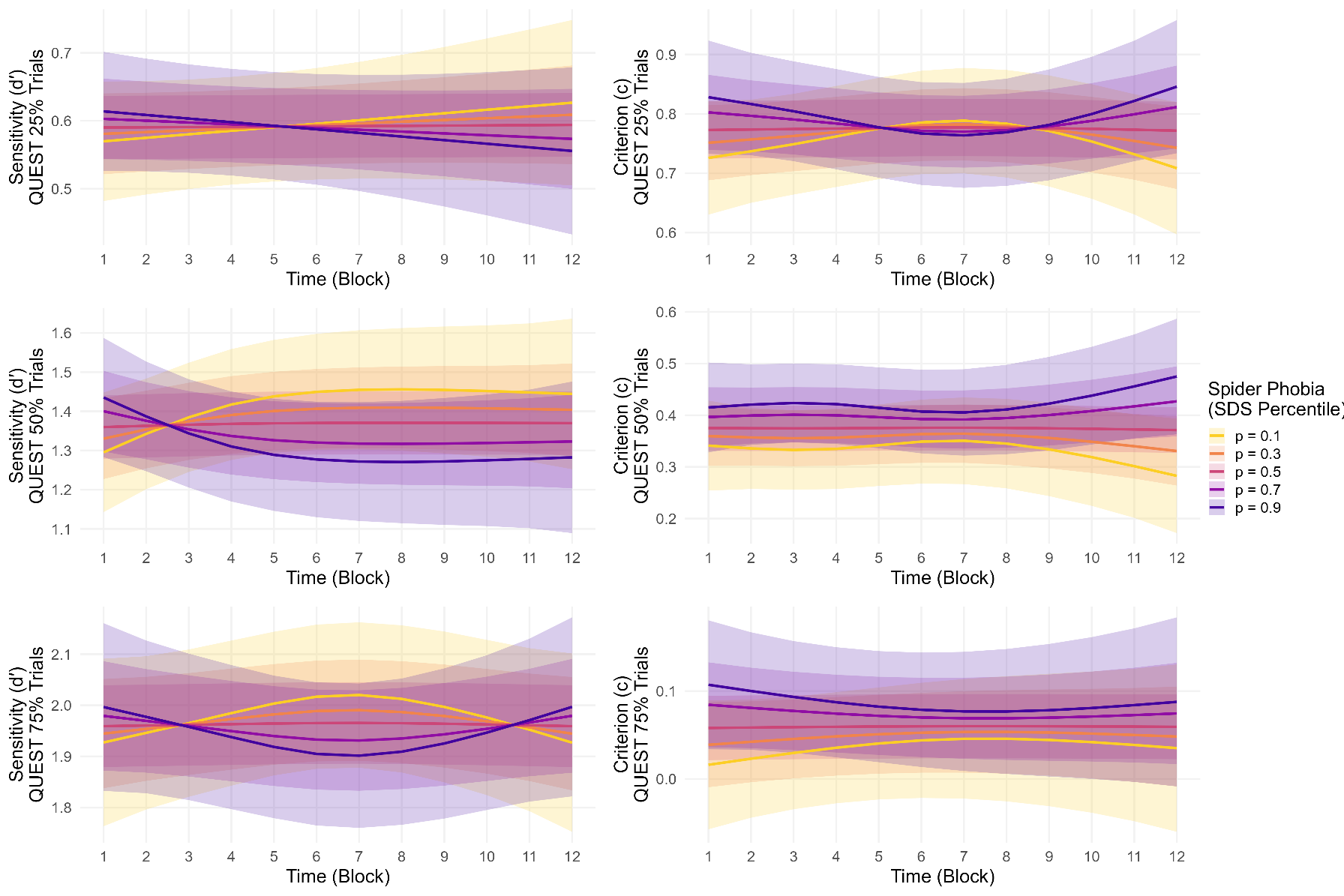


**Figure S21.** Predicted trajectories of sensitivity (d′, left) and criterion (c, right) across experimental blocks in the flowers group, separated by QUEST difficulty levels: 25% (top), 50% (middle), and 75% (bottom). Lines reflect spider phobia percentiles (10th–90th); shaded areas show 95% confidence intervals.

**8.2 Model diagnostics**

Model diagnostics were generated using the gam.check() function from the mgcv package [15], and the resulting diagnostic plots are shown in Figures S22–27. Overall, the diagnostics indicate no major problems with model fit: there is no evidence of heteroskedasticity or clear model misspecification. Although some models show minor departures from residual normality (e.g. S22 and S27), these deviations are small and unlikely to meaningfully affect inference, as mixed-effects models are generally robust to mild violations of normality [12].

We next moved on to the diagnostics for the non-linear estimation terms. Once again, note that the k-index *p*-values are simulation-based and may differ slightly across runs. For one model (criterion–QUEST 50%: k′ = 4, edf = 2.79, k-index = 0.97, p = .090), the k-index was close to 1 and the p-value was non-significant, suggesting that the chosen basis dimension was adequate. However, all the rest had lower k-index values and significant p-values (criterion–QUEST 25%: k′ = 4, edf = 3.02, k-index = 0.95, p = .010; criterion–QUEST 75%: k′ = 4, edf = 2.75, k-index = 0.85, p < .001; d′–QUEST 25%: k′ = 4, edf = 1.90, k-index = 0.95, p = .005; d′–QUEST 50%: k′ = 4, edf = 1.00, k-index = 0.90, p < .001; d′–QUEST 75%: k′ = 4, edf = 1.00, k-index = 0.88, p < .001), suggesting that the basis dimension might have been too restrictive.

As a robustness check, we re-fit the models with significant k-test results (i.e. all but criterion–QUEST 50%) and doubled the number of allowed basis functions (i.e. k = 10). Based on these updated models, the k-test diagnostics indicated very similar model fit to the original models (criterion–QUEST 25%: k′ = 9, edf = 3.36, k-index = 0.95, p = .013; criterion–QUEST 75%: k′ = 9, edf = 3.74, k-index = 0.85, p < .001; d′–QUEST 25%: k′ = 9, edf = 1.98, k-index = 0.95, p < .001; d′–QUEST 50%: k′ = 9, edf = 1.00, k-index = 0.90, p < .001; d′–QUEST 75%: k′ = 9, edf = 1.00, k-index = 0.88, p < .001). Additionally, the overall findings of the direction and significance of the interaction between block number and spider phobia in these models were the same (criterion–QUEST 25%: k′ = 9, edf = 3.36, F = 4.33, p = .003; d’–QUEST 25%: k′ = 9, edf = 1.98, F = 1.14, p = .330; d’–QUEST 50%: k′ = 9, edf = 1.00, F = 0.38, p = .536; d’–QUEST 75%: k′ = 9, edf = 1.00, F = 0.54, p = .462), with the exception of the criterion QUEST 75% model, where the effect changes from a marginally insignificant effect to a significant one (criterion–QUEST 75%: k′ = 9, edf = 3.74, F = 2.97, p = 0.028). Because the k-test diagnostics do not clearly favor either model, this positive result is at best not very robust. Overall, because increasing the number of basis dimensions did not change the pattern of results, the main findings observed are unlikely to reflect an overly restrictive basis dimension.

**
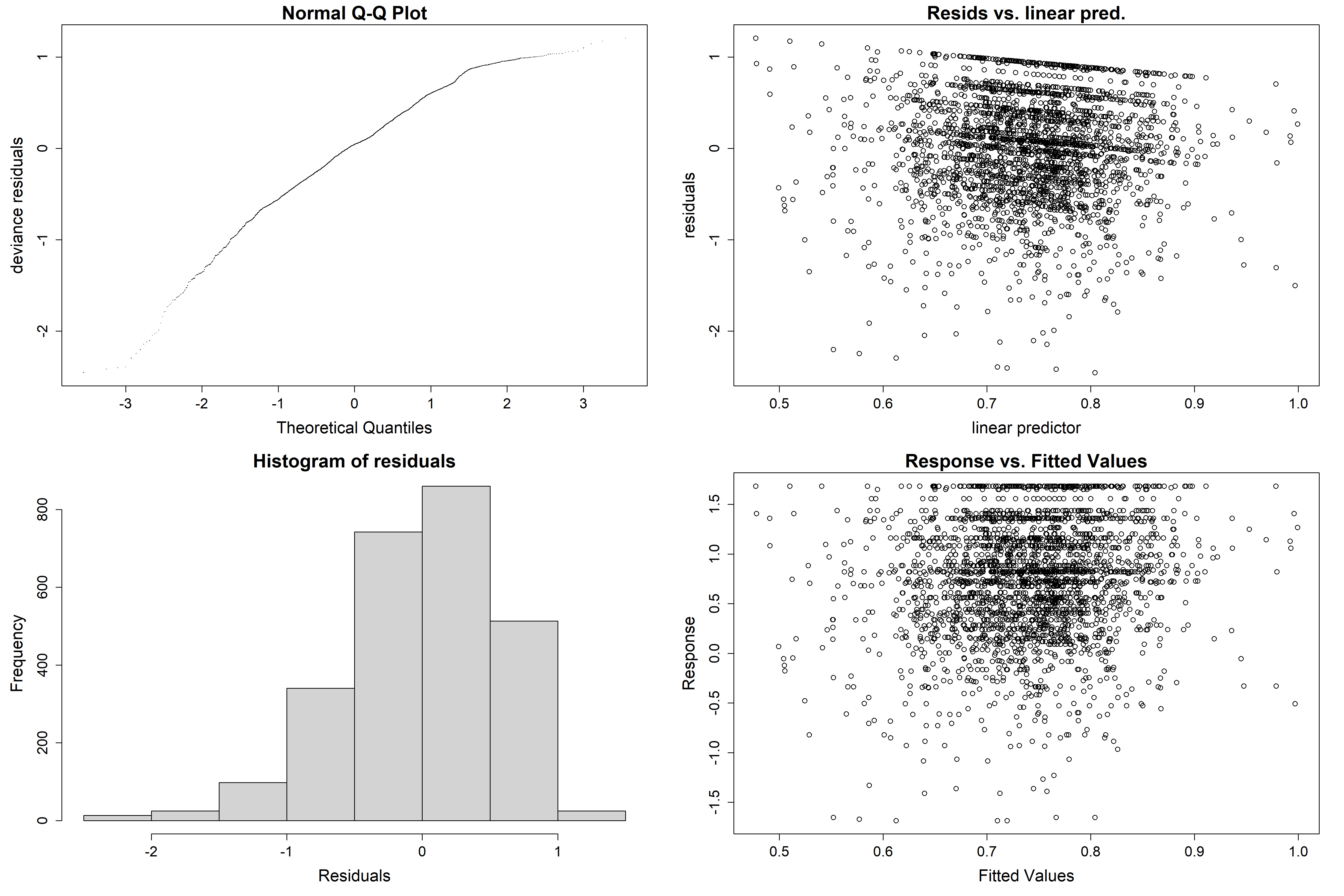
**

**Figure S22.** Model diagnostic plots for the generalized additive mixed model (GAMM) of criterion (c) at QUEST 25%. The panels display (top-left) a Q–Q plot of deviance residuals, (top-right) residuals versus linear predictor, (bottom-left) a histogram of residuals, and (bottom-right) observed responses versus fitted values.

**
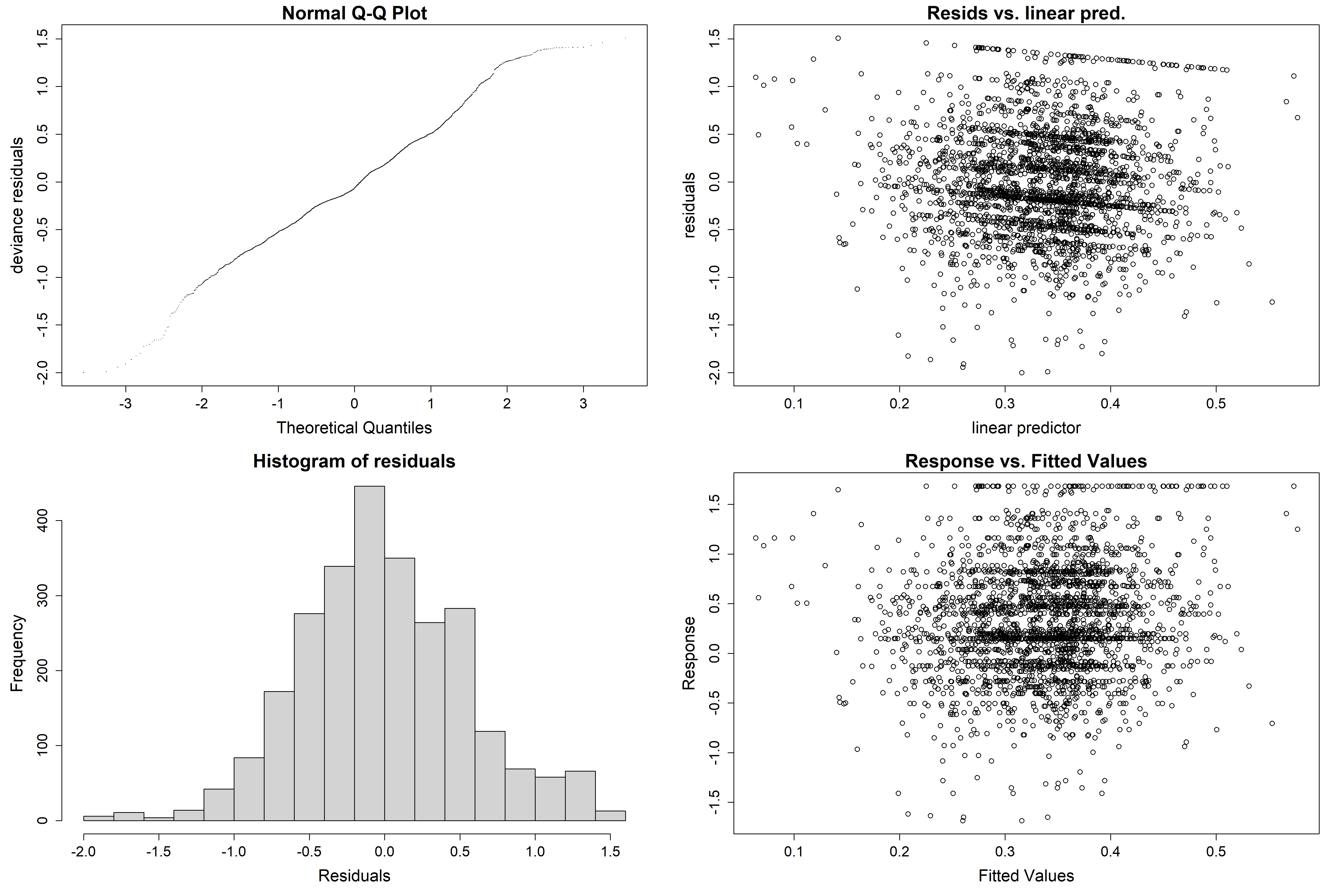
**

**Figure S23.** Model diagnostic plots for the generalized additive mixed model (GAMM) of criterion (c) at QUEST 50%. The panels display (top-left) a Q–Q plot of deviance residuals, (top-right) residuals versus linear predictor, (bottom-left) a histogram of residuals, and (bottom-right) observed responses versus fitted values.

**
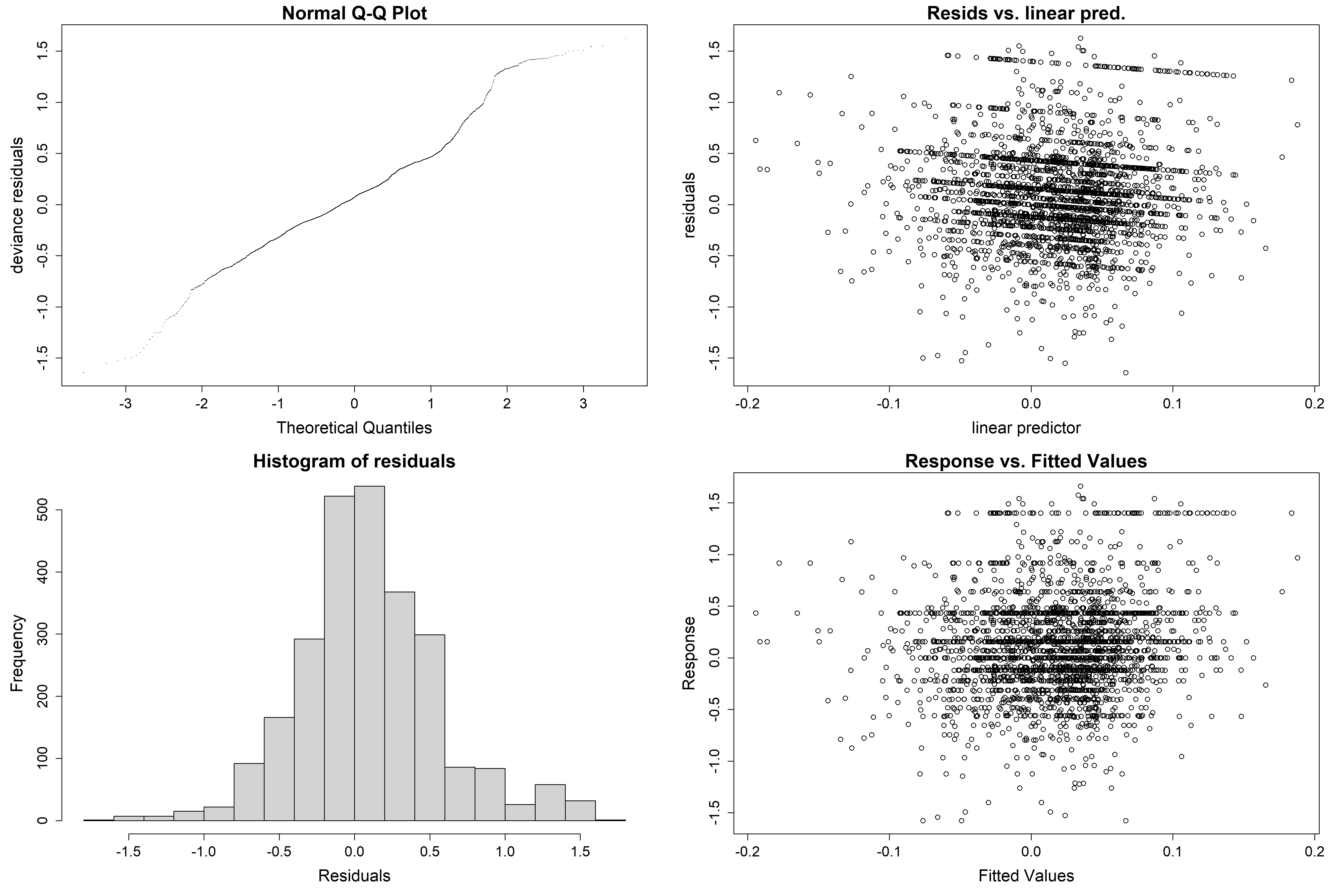
**

**Figure S24.** Model diagnostic plots for the generalized additive mixed model (GAMM) of criterion (c) at QUEST 75%. The panels display (top-left) a Q–Q plot of deviance residuals, (top-right) residuals versus linear predictor, (bottom-left) a histogram of residuals, and (bottom-right) observed responses versus fitted values.

**
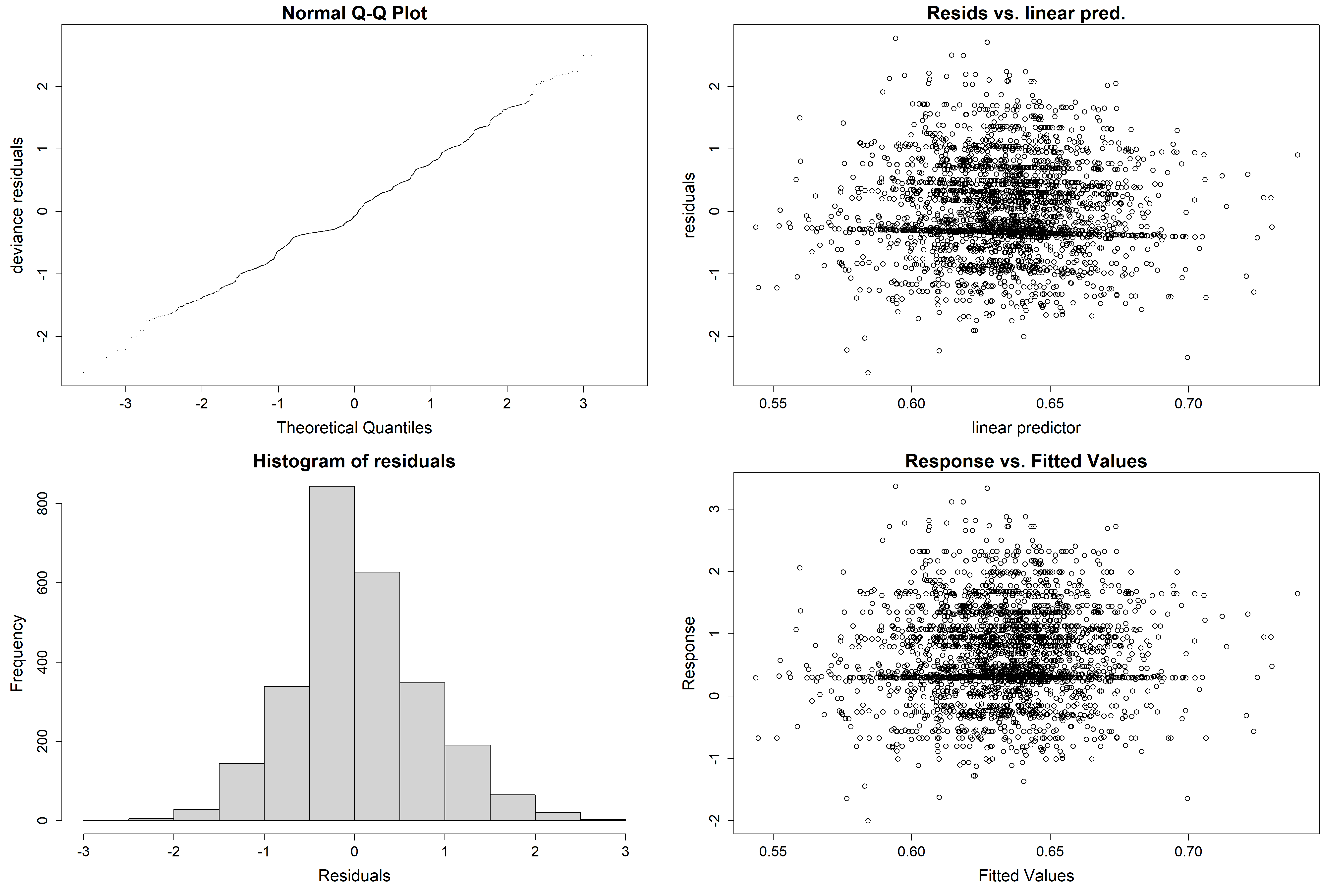
**

**Figure S25.** Model diagnostic plots for the generalized additive mixed model (GAMM) of sensitivity (d′) at QUEST 25%. The panels display (top-left) a Q–Q plot of deviance residuals, (top-right) residuals versus linear predictor, (bottom-left) a histogram of residuals, and (bottom-right) observed responses versus fitted values.

**
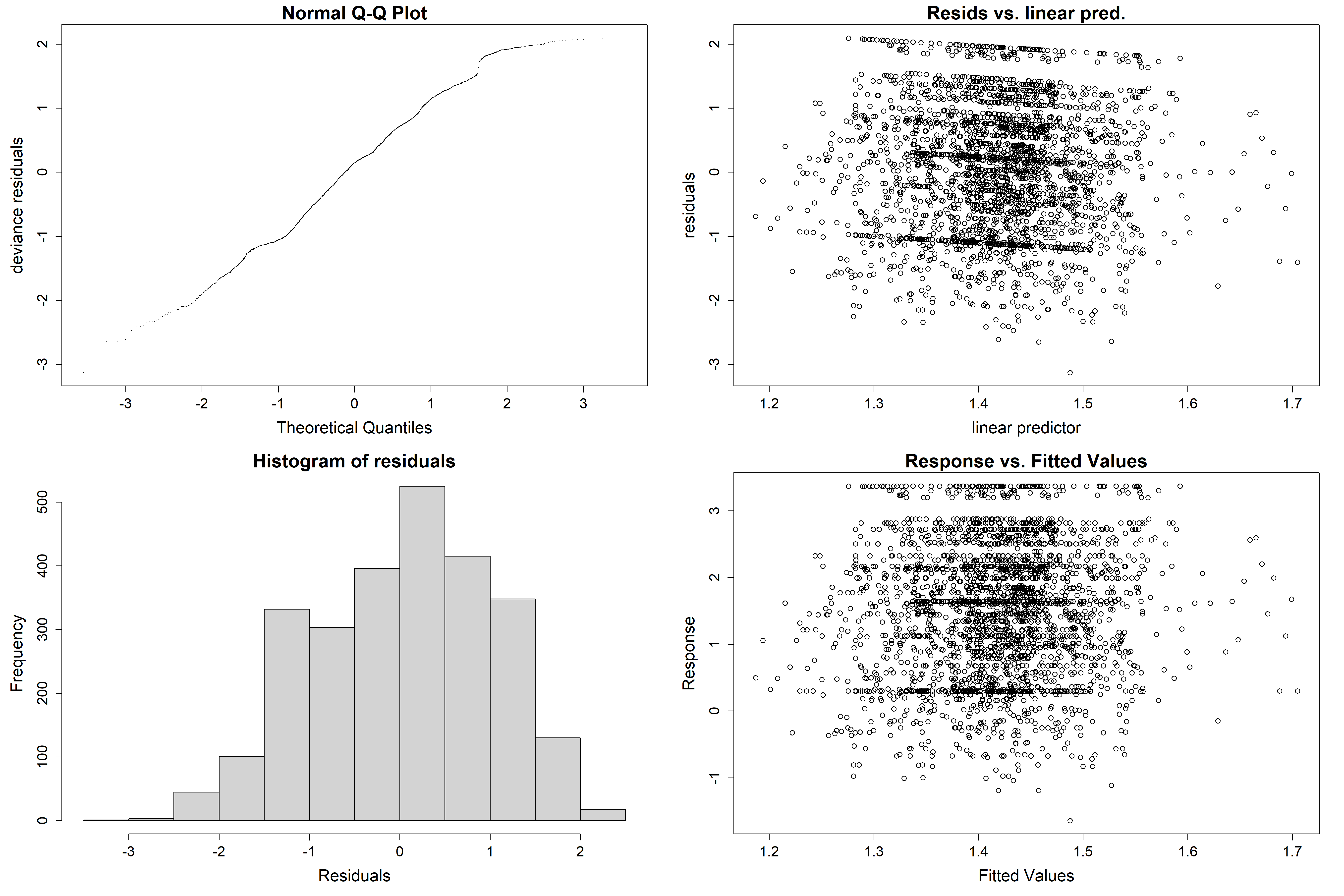
**

**Figure S26.** Model diagnostic plots for the generalized additive mixed model (GAMM) of sensitivity (d′) at QUEST 50%. The panels display (top-left) a Q–Q plot of deviance residuals, (top-right) residuals versus linear predictor, (bottom-left) a histogram of residuals, and (bottom-right) observed responses versus fitted values.

**
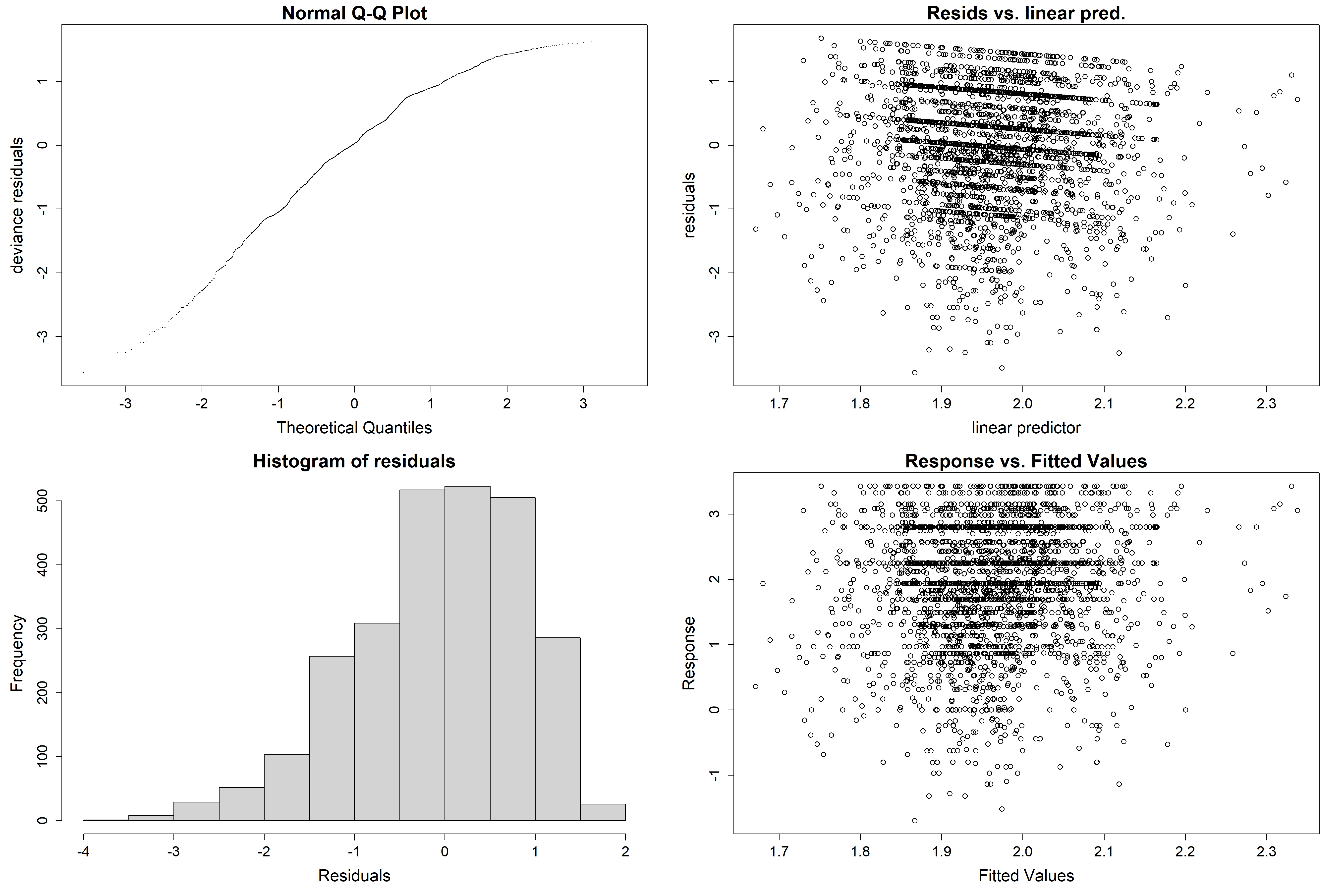
**

**Figure S27.** Model diagnostic plots for the generalized additive mixed model (GAMM) of sensitivity (d′) at QUEST 75%. The panels display (top-left) a Q–Q plot of deviance residuals, (top-right) residuals versus linear predictor, (bottom-left) a histogram of residuals, and (bottom-right) observed responses versus fitted values.

**References**

1. Kafadar, E. et al. Conditioned hallucinations and prior overweighting are state-sensitive markers of hallucination susceptibility. Biol. Psychiatry 92, 772–780 (2022).
2. Schmack, K., Burk, J., Haynes, J. D. & Sterzer, P. Predicting subjective affective salience from cortical responses to invisible object stimuli. Cereb. Cortex 26, 3453–3460 (2016).
3. Zuur, A. F., Ieno, E. N., Walker, N. J., Saveliev, A. A. & Smith, G. M. Mixed effects models and extensions in ecology with R. (Springer, New York, 2009).
4. R Core Team. R: A language and environment for statistical computing, version 4.4.1 (R Foundation for Statistical Computing, Vienna, 2024); https://www.R-project.org/
5. Lüdecke, D., Ben-Shachar, M. S., Patil, I., Waggoner, P. & Makowski, D. performance: an R package for assessment, comparison and testing of statistical models. J. Open Source Softw. 6, 3139; 10.21105/joss.03139 (2021).
6. Hartig, F. DHARMa: Residual diagnostics for hierarchical (multi-level / mixed) regression models. R package version 0.4.7. https://CRAN.R-project.org/package=DHARMa (2022).
7. Bates, D., Mächler, M., Bolker, B. & Walker, S. Fitting linear mixed-effects models using lme4. J. Stat. Softw. 67, 1–48; https://doi.org/10.18637/jss.v067.i01 (2015).
8. Christensen, R. H. B. ordinal: Regression models for ordinal data, version 2019.12–10; https://doi.org/10.32614/CRAN.package.ordinal (2019).
9. Bürkner, P. C. brms: An R package for Bayesian multilevel models using Stan. J. Stat. Softw. 80, 1–28; https://doi.org/10.18637/jss.v080.i01 (2017).
10. Bürkner, P. C. Advanced Bayesian multilevel modeling with the R package brms. R J. 10, 395–411 (2018).
11. Stan Development Team. RStan: The R interface to Stan, version 2.32.7; https://mc-stan.org/ (2025).
12. Schielzeth, H. et al. Robustness of linear mixed-effects models to violations of distributional assumptions. Methods Ecol. Evol. 11, 1141–1152 (2020).
13. Pustejovsky, J. E. clubSandwich: Cluster-robust (sandwich) variance estimators with small-sample corrections, version 0.6.1; https://CRAN.R-project.org/package=clubSandwich/ (2025).
14. Venables, W. N. & Ripley, B. D. Modern applied statistics with S, 4th edn (Springer, New York, 2002); https://www.stats.ox.ac.uk/pub/MASS4/
15. Wood, S. N. Fast stable restricted maximum likelihood and marginal likelihood estimation of semiparametric generalized linear models. J. R. Stat. Soc. B 73, 3–36 (2011).
